# Mandated Sick Pay: Coverage, Utilization, and Crowding-in

**DOI:** 10.1093/jeea/jvaf008

**Published:** 2025-02-22

**Authors:** Johanna Catherine Maclean, Stefan Pichler, Nicolas R Ziebarth

**Affiliations:** George Mason University, United States; University of Groningen, Netherlands; ZEW Mannheim and University of Mannheim, Germany

## Abstract

Using the National Compensation Survey from 2009 to 2022 and difference-in-differences methods, we find that state-level sick pay mandates are effective in broadening access to paid sick leave for U.S. workers. Increases in sick pay coverage reach 30 percentage points from a 63% baseline 5 years post-mandate. Mandates have more bite in jobs with low pre-mandate coverage. Further, mandates reduce inequality in access to paid sick leave substantially, both across and within firms. COVID-19 reinforced existing upward trends in coverage and take-up. Five years post mandate, sick leave use has linearly increased to 2.4 days per year for marginal jobs. Finally, we find *crowding-in* of non-mandated benefits, which we label “job upscaling” by firms to differentiate jobs and attract labor.

## 1. Introduction

For decades, the design of social insurance systems has been a core research field in economics (Card, Kluve, and Weber 2017; Nekoei and Weber 2017; Powell and Seabury 2018; Fadlon and Nielsen 2019; Johnson 2020). Economists have studied questions such as: What are the consequences when the government mandates employers to pay minimum wages or provide benefits such as paid parental leave (Summers 1989; Lalive et al. 2014; Cengiz et al. 2019)? How effective are such mandates and what is the impact on employers and employees (Gruber 1994; Ruhm 1998)? Are there unintended consequences (Bailey et al. 2019)? This paper empirically studies these questions for U.S. sick pay mandates over the last 15 years. In particular, we estimate the impact of state-level sick pay mandates on (inequality in) job coverage, utilization (“moral hazard”), labor costs, and non-mandated fringe benefits (‘spillovers’).

Of the countries in the Organization for Economic Cooperation and Development (OECD), four do not mandate universal access to short-term paid sick leave for employees: Canada, Japan, Korea, and the United States. Traditionally, in the United States, employers have voluntarily provided paid sick leave. This is true even after the COVID-19 pandemic that resulted in the first federal, but temporary, emergency sick pay provision (H.R.6201—Families First Coronavirus Response Act 2020; Jelliffe et al. 2021). Nevertheless, in March of 2022, 23% of all private sector jobs *lacked* paid sick leave. Further, there exists substantial inequality in paid sick leave access across types of jobs. While 98% of private sector jobs in the insurance industry have paid sick leave, only 53% of all jobs in accommodation and food services offer this benefit. In the bottom wage quintile of jobs, 38% provide sick pay, but in the top quintile, 96% provide sick pay (Bureau of Labor Statistics 2023a). As a result, Congress has debated for decades whether to pass the Healthy Families Act (2023), which contains a sick leave provision similar to the ones studied in this paper.^1^ Because of a lack of bipartisan support at the federal level, as of writing, 15 states, DC and dozens of cities have passed sick pay mandates.

Although all but four OECD countries guarantee universal access to paid sick leave for short-term sickness, the design of the schemes varies substantially across countries (Heymann et al. 2010; OECD 2010; Raub et al. 2018; OECD Policy Responses to Coronavirus 2020). In Europe, short-term sick leave is typically organized via employer mandates, meaning a law requires employers to provide benefits. Further, long-term sick pay (called “medical leave” in the United States) is typically provided by social insurance institutions where employees and/or employers must apply to take-up benefits. The latter is also true for disability insurance, which provides social insurance benefits when employees are permanently work disabled. Pichler and Ziebarth (2024) provide an overview and a categorization of such benefits.

Eligibility periods and replacement levels of employer-provided short-term sick leave vary across OECD countries as well. Germany has one of the most generous systems and provides a 100% replacement rate for up to 6 weeks per sickness spell (Ziebarth and Karlsson 2010, 2014). Sweden offers 14 days of employer-provided sick leave at a minimum replacement rate of 80% and has a waiting period of 1 day—that is, there is no mandated wage replacement for the first day of a spell to reduce shirking behavior (Hesselius, Nilsson, and Johansson 2009). The countries require doctors’ notes after three (Germany) and seven (Sweden) consecutive sick days. Doctors' notes are not required in the United States as it would put undue burden on those with high deductibles or a lack of access to healthcare professionals. However, employees must notify their employers when sick, both in Europe and the United States.

While the design of European sick leave schemes resembles that of unemployment insurance, in the United States, paid sick leave resembles saving accounts (Feldstein and Altmann 2007): Employers are mandated to maintain sick leave accounts with a running balance for each employee’s sick pay credit. Employees thus have the right to “earn” 1 hour of paid sick time (at 100% of the wage) per 30–40 hours of working time. Sick pay credit is thus individualized; earned and unused sick time accumulates over the course of a year, and employees can take it whenever needed. Unused sick time rolls over to the next year. Note that employees can also take sick time to take care of sick children or for healthcare services.

This paper uses a firm-level survey by the Bureau of Labor Statistics (BLS) to estimate the first-order effects of U.S. state-level sick pay mandates on (a) the probability that jobs provide paid sick leave, (b) the use of both unpaid and paid sick leave, (c) labor costs, and (d) a range of non-mandated fringe benefits such as paid vacation or group insurance policies that employers could systematically reduce in response to the mandates. We also assess whether hours worked and paid systematically change in response to the mandates.

Existing papers have also estimated effects on coverage and take-up using the National Health Interview Survey, American Community Survey, and Current Population Survey; however, they rely on employee self-reports, which may suffer from response and recall biases (Stearns and White 2018; Callison and Pesko 2022; Slopen 2024).^2^ To derive policy recommendations, the effect of gaining access on paid and unpaid sick leave use and on labor costs is crucial. Our data contain both hourly paid and unpaid use as well as labor costs linked to sick leave, calculated by the BLS. Moreover, to assess possible unintended consequences, testing whether mandates crowd-out non-mandated fringe benefits is important. Our data include a range of non-mandated benefits and allow for such estimates.

To this end, we use restricted-access data from the National Compensation Survey (NCS) at the firm-job level from 2009 to 2022, coupled with difference-in-differences (DD) models. We use the Callaway and Sant’Anna (2021) (CS) DD estimator that is robust to heterogeneous and dynamic treatment effects under staggered policy adoption over states and time. We also plot CS event studies. NCS data are specifically designed to measure employee compensation and employer costs—indeed, the U.S. government uses the NCS to adjust federal employee compensation.

**Figure 1.**
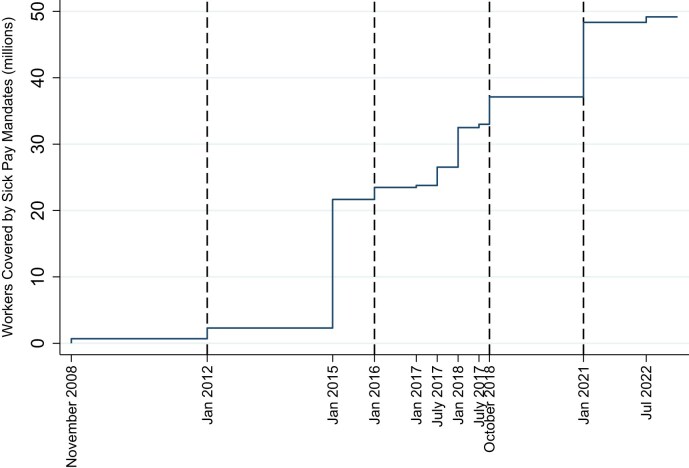
Number of employees covered by state-level sick pay mandates. Source: Bureau of Labor Statistics (2023e). Own data collection and illustration. The figure shows the number of private sector employees covered by sick pay mandates between 2009 and 2022 in D.C., Connecticut, California and Massachusetts, Oregon, Vermont, Arizona, Washington, Maryland, Rhode Island, and New Jersey. Employees in city and county level jurisdictions with mandates are not included, and neither are they in our models.

Figure 1 illustrates that how the number of U.S. private sector employees covered by state-level sick pay mandates had increased from 2009 to 2022, namely from half a million in 2009 to more than 48 million in 2022, representing about a third of all U.S. employees in 2022. From 2009 to 2022, 13 U.S. states implemented sick pay mandates.^3^ For four states (California, Connecticut, Massachusetts, and Oregon), we observe at least five post-mandate years.

We find that state-level mandates are effective in increasing sick pay access. They also reduce inequality in access to paid sick leave across jobs. Within the first 5 years after the mandates’ implementation, on average, the probability that a job provides paid sick leave increases by 20 percentage points (ppt) or 32% from a pre-mandate level of 63%. The increase grows over time during the first 4 years and then plateaus. Access to this benefit enables more employees to take sick days: On average, paid sick leave use increases by a significant 3.9 hours per year. Scaling this average increase by the rise in coverage implies that employees in newly covered jobs take about two additional sick days per year. Unpaid sick leave use—mandates also compel coverage of unpaid leave—increases by a significant 0.85 hours per year for newly covered jobs. These relatively modest increases in sick leave use even five years into the mandates’ implementation either suggest reductions of infections at the workplace and/or the absence of widespread shirking behavior, consistent with existing evidence and in line with incentive-compatibility of individualized sick pay credit (Pichler, Wen, and Ziebarth 2020; Cronin, Harris, and Ziebarth 2022; Andersen et al. 2023).

Sick leave costs for firms increase by a significant 6 cents per hour worked or by, scaled, 31 cents in jobs that newly provide sick pay. Finally, consistent with positive cascading effects after the introduction of minimum wages, we find positive spillover effects on non-mandated benefits such as short- and long-term disability insurance policies, life insurance, and dental plans. We dub this finding “job upscaling.” In line with responses from employer surveys, we interpret job upscaling as firm efforts to differentiate themselves (or the jobs) from competitors (or part-time jobs) to attract labor.

This paper contributes to several literatures. First and foremost, the paper contributes to the economics literature on sick leave. In the United States, the literature has begun to develop,^4^ but it has a much longer tradition in Europe.^5^ One general finding in the literature is that labor supply is elastic with respect to the benefit level (“moral hazard”); the elasticity is estimated to lie around −1 (Johansson and Palme 1996, 2002, 2005; Ziebarth and Karlsson 2010, 2014; De Paola, Scoppa, and Pupo 2014; Fevang Markussen, and Røed 2014; Kanninen, Böckerman, and Suoniemi 2022). Further, more generous sick leave reduces the spread of infectious diseases and relapses (Pichler and Ziebarth 2017; Stearns and White 2018; Pichler, Wen, and Ziebarth 2020; Marie and Vall-Castello 2023; Pichler, Wen, and Ziebarth 2021; Andersen et al. 2023). Adams-Prassl et al. (2023) find in survey experiments that providing information on the positive health externality of paid sick leave increases support for a public provision of sick pay.

The rich European literature also finds that sick leave and other social insurance programs are complements (Fevang, Hardoy, and Røed 2017); that sick leave use is lower during probation periods (Ichino and Riphahn 2005), that women take more sick leave than men (Ichino and Moretti 2009; Herrmann and Rockoff 2012), that culture (Ichino and Maggi 2000) and social norms (Bauernschuster et al. 2010) matter, that peer effects at the workplace play a role (Hesselius, Nilsson, and Johansson 2009), that union members are more prone to taking sick leave (Goerke and Pannenberg 2015), that "compulsory dialogue meetings" do not reduce leave taking for short-term (Alpino et al. 2022) but for long-term sick leave (Markussen, Rød, and Schreiner 2018)—as does gatekeeping through physician certification (Markussen and Røed 2017)—while a lower unemployment rate (Nordberg and Røed 2009), and higher marginal taxes increase sick leave use (Dale-Olsen 2013).

Further, recent work using Latin American administrative data find that Brazilian employers respond to sickness spells by increasing hiring, but the hiring effect is much larger for maternity leave (Schmutte and Skira 2024). Barone (2023) estimates a structural model of optimal sick leave using Chilean administrative data and variation in economic incentives by day of the week. She finds that the replacement rate of the optimal, welfare improving, sick pay scheme decreases in the spell duration.

This paper also contributes to research on labor market inequalities (Card, Heining, and Kline 2013; Maestas et al. 2018; Song et al. 2019) and paid family (parental) leave (Ruhm 1998; Lalive et al. 2014; Dahl et al. 2016; Baum and Ruhm 2016; Brenøe et al. 2023).^6^ Finally, the paper also contributes to research on disability insurance and workers’ compensation (Staubli 2011; Maestas, Mullen, and Strand 2013; Powell and Seabury 2018; Dahl and Gielen 2021; Cao, Fischer, Geyer, and Ziebarth 2022; Hallter, Staubli, and Zweimüller 2023) as well as the economics of employer mandates (Summers 1989; Gruber 1994) and optimal social insurance more generally (Chetty and Finkelstein 2013; Kolsrud et al. 2018).

## 2. U.S. Sick Pay Mandates

Paid sick leave was an integral part of the first social insurance system in the world. The Sickness Insurance Law of 1883 implemented federally mandated employer-provided health insurance in Germany, which covered up to 13 weeks of paid sick leave along with healthcare. Paid sick leave—insurance against wage losses due to health shocks—was a crucial element of health insurance at that time. Given the limited availability of medical treatments in the 19th century, expenditures for paid sick leave initially accounted for more than half of all health insurance costs (Busse and Blümel 2014). Subsequently, other European countries also implemented sick leave mandates. Today, although the generosity varies, every European country provides universal access to paid sick leave for employees.

In the United States, Senator Theodore Kennedy spearheaded the first legislation for a federal sick pay mandate—the *Healthy Families Act*. First introduced to the U.S. Congress in 2005, the bill was reintroduced in 2023 after several failed attempts at passage (Healthy Families Act 2023). In the meantime, numerous U.S. cities and states have passed similar sick pay mandates within their jurisdictions. San Francisco was the first locality to implement a mandate in 2007, increasing coverage rates above 90% among employees (Colla et al. 2014). In the following years, based on widespread voter support—opinion polls suggest that 75% of Americans support sick pay mandates, with majority support across party affiliation (National Paid Sick Days Study 2010; HuffPost/YouGov 2013)—a wave of cities and states adopted sick leave legislation. As of writing, 15 states, D.C. and 20 cities and counties (including Chicago, New York City, Philadelphia, Portland, and Seattle) have passed sick pay mandates; see A Better Balance (2024) and National Partnership for Women and Families (2024). This paper uses data up to and including 2022 and variation from 12 states.

Moreover, in response to the COVID-19 pandemic, in March of 2020, Congress passed a bipartisan *Families First Coronavirus Response Act (FFCRA)*. The Act mandated up to 2 weeks of COVID-19 related emergency sick leave for employees in private firms with 50–500 employees (H.R.6201—Families First Coronavirus Response Act 2020). This emergency provision has now expired. Many other countries around the world also enacted legislation to bolster sick pay access due to COVID-19; see OECD Policy Responses to Coronavirus (2020).

*FMLA*. In the United States, the *Family and Medical Leave Act (FMLA)* is the only current federal law related to leave. FMLA stipulates employees’ rights to take *unpaid* leave in case of pregnancy, own sickness, or sickness of a family member. The Act applies to employees who work at least 1,250 hours per year for a firm with at least 50 employees (Waldfogel 1999). Jorgensen and Appelbaum (2014) estimate that 44% of private sector employees are eligible for FMLA. The state-level sick pay mandates analyzed in this paper provide employees with the right to take paid and also *unpaid* sick leave. This entails dismissal protection while on sick leave. That is, although U.S. employment is overwhelmingly at will and employees can be terminated without reason or warning, employers cannot terminate employees for taking sick leave.

*Sick Time Credit and Accrual Rates*. Online Appendix Table A.1 provides a summary of all U.S. state-level mandates enacted at the time of writing. While the details of the mandates differ, all mandates are employer mandates, meaning that the law mandates employers to provide sick leave as follows: Employees gain the right to “earn” sick time credit of typically 1 hour for every 30–40 hours worked. This credit implies paid sick leave at a 100% wage rate when taken. If unused, the sick time credit rolls over to the next calendar year. Because employees must first accrue credit, most mandates stipulate a 90-day accrual period before employees can start taking paid sick leave. Further, there exist waiting periods for newly hired employees similar to European countries, meaning that employees cannot take sick leave immediately after starting a new job. Finally, several states exempt small firms but then typically mandate them to provide *unpaid* sick leave (Massachusetts Attorney General’s Office 2016).

All state-level mandates stipulate individual credit accounts and have similar structures, framed after the *Healthy Families Act* (Healthy Families Act 2023). While accrual rates and waiting periods differ slightly,^7^ the design is otherwise relatively homogeneous. Thus, while we compare the effects across states, we do not differentiate by mandate generosity in our empirical specifications. That is, we primarily study the effects on the extensive, not the intensive, margin.

*Qualifying Reasons*. As seen in Online Appendix Table A.1, qualifying reasons for sick leave are own sickness or sickness of a dependent child, spouse/partner, and sometimes additional family members (e.g., parents). Note that, in contrast to most European schemes, employers cannot require doctors’ notes. Rather, moral hazard is mainly contained through the individualized sick time credits in combination with relatively restrictive accrual rates of 1 hour credit per 30–40 hours worked.^8^ Note that *none* of the mandates levy an explicit employer or employee tax to fund the sick days. Instead, benefits are funded entirely through work credit and an employer mandate, as described above.

*Workplace Notification*. Firms must post notifications about paid sick leave rights at the workplace. Online Appendix Figure A.1(a) shows a sick time notice that complies with Massachusetts’ workplace poster requirements (Commonwealth of Massachusetts 2019). Alternatively, firms could post notices as in Online  Appendix Figure A.1(b) that include *all* employee rights (Industrial Commission of Arizona 2019).

*Substate Mandates*. In addition to states, dozens of cities have passed sick pay mandates since 2009; see A Better Balance (2024) for an overview. This paper focuses on the state-level mandates and disregards all sub-state mandates due to the geographic information in our data. As detailed below, the main reason is that county identifiers do not map to the city-level mandate boundaries, plus the county-level data suffer from small, non-representative, sample sizes. We routinely drop counties that adopted mandates and counties that include city-level mandates. Note that these counties or cities passed mandates *prior* to their states.^9^ Whenever state and substate mandates coexist, legal complexities arise: When states pass mandates, existing substate laws are typically preempted; for example, the 13 New Jersey city laws that existed prior to the state law (Title 34. Chapter 11D. (New) sick leave 1–11). However, pre-emption is not always the case, especially not when city laws are passed *after* the state law and are more comprehensive. Because we focus on state-level mandates, we circumvent the legal complexities of this institutional city-state-interplay.

*Lawsuits*. Sick pay mandates have been challenged through the court system, mostly by business groups seeking to have the laws overturned. For example, Airlines for America have sued the states of Massachusetts and Washington to seek an exemption from the law, arguing that the law would adversely affect their carrier prices, routes, and services (Bloomberg BNA—Workplace Law Report 2018). As another example, the Massachusetts Supreme Judicial Court ruled that sick pay does not constitute wages, which implies that firms are not liable if they do not pay out unused sick days to employees (Kaczmarek 2018). In the empirical specifications, we do not differentiate by whether a lawsuit is pending anywhere at a given time for a specific jurisdiction.

*Discrimination*. One possible unintended consequence of the mandates is that firms may now discriminate against employees based on observable factors that firms believe are correlated with sick leave use. Federal anti-discrimination law may limit such potential discrimination. Moreover, compared to other mandated benefits, for example, workers compensation or health insurance, sick leave mandates are relatively minor mandates in terms of costs to employers. Thus, mandate-induced discrimination is likely negligible but we cannot rule it out. As this paper primarily estimates the impact of the mandates on firm-level provision of benefits, discrimination in recruiting is outside the scope of this paper. In terms of the impact on sick leave use, we would expect such (illegal) hiring practices to mute mandate effects. Our intent-to-treat (ITT) estimates would then reflect lower bounds compared to a counterfactual without changes in the employee composition.

## 3. National Compensation Survey

The NCS is designed to provide a detailed picture of wage and non-wage compensation in the United States. The data are used to produce government statistics on a wide range of compensation and labor cost items. The data are also used to adjust wages for federal employees.

### 3.1 Sampling and Sample Selection

The NCS is a rotating panel of firms,^10^ where firms typically stay in the sample for 3–5 years. Further, the NCS is nationally representative at the firm-job level. Throughout our analysis, we use BLS survey weights to provide nationally representative estimates at the job level.^11^ We use the restricted access version of the NCS, which is collected and maintained by the BLS. Importantly, the restricted access version includes county identifiers, which allows us to match state-level mandates to the data and drop fully or partially treated counties; see Section 2.

#### Sampling.

In the NCS, random sampling is first carried out at the firm level. The BLS defines firms as “a single economic unit that engages in one, or predominantly one, type of economic activity” (Bureau of Labor Statistics 2023b). Second, for every job within each firm, the NCS collects information on compensation and benefits at the *job* level (Bureau of Labor Statistics 2023b).^12^ The BLS selects firms and jobs within firms probabilistically. Within a selected firm, four to eight jobs are probabilistically sampled from a list of employees provided by the firm. Thus, in the NCS, a job is an employee or a group of employees within a sampled firm with the same job. Please see the NCS documentation for full details (Bureau of Labor Statistics 2023c). In the manuscript, for stylistic reasons, we use the terms “firm,” “employer,” and “establishment” as synonyms.

The human resource administrator of each firm then provides detailed information to the BLS field economists on a range of wage and non-wage benefits, including paid sick leave as well as paid and unpaid sick leave use. Because the information uses firm-level administrative records, response error due to, for example, employees being unaware of their benefits is minimized. Further, these data allow us to explore potential spillovers from sick leave mandates to non-mandated benefits such as paid vacation or parental leave.

#### Interview Timing and Reform Coverage.

In principle, the NCS is a quarterly survey. However, we focus on the first quarter responses at the end of March; this is because the BLS only provides information from this interview for many benefits, including paid sick leave. This implies that we leverage six annual waves of post-mandate data for four states (California, Connecticut, Massachusettes, and Oregon), five annual waves of post-mandate data for another four states (Arizonia, Maryland, Vermont, and Washington), four waves for two states (New Jersey and Rhode Island), and two waves for two states (CO and NY).

#### Stock versus Flow Measures.

One can distinguish between stock and flow measures in the NCS. The stock measures (such as access to paid sick leave) refer to the status quo at the time of the first quarter interview in March. The flow measures (such as sick leave utilization) generally refer to *the past 12 months*; that is, from April of the previous year to March of the survey year.^13^ Finally, note that we only observe the total average number of sick hours taken in the past 12 months, but do not see *when* specifically these hours were taken.

#### Sample Selection.

In our main analysis, we leave the micro-data at the firm-job level and restrict the sample to private sector firms (as the mandates only apply to the private sector). Table 1 reports the summary statistics. In our main sample, we have 443,740 observations at the firm-job level for the years 2009–2022. Using the Consumer Price Index, we convert all monetary values to 2019 U.S. dollars (Bureau of Labor Statistics 2024).

**Table 1.** Descriptive statistics, NCS.

	All	Treated states, pre-mandate	Control states	Normalized difference
***Main outcomes***				
Sick leave offered (binary)	0.632	0.626	0.597	0.1618
Paid sick hours taken (hours p.a.)	16.79	17.99	15.42	0.1727
Unpaid sick hours taken (hours p.a.)	0.153	0.135	0.132	0.0007
Sick leave costs ($per hour worked)	0.275	0.338	0.233	0.2129
***Job characteristics***				
Full-time employment (binary)	0.738	0.724	0.742	0.0061
Part-time employment (binary)	0.262	0.276	0.258	0.0061
Non-unionized (binary)	0.924	0.899	0.932	0.0775
Unionized (binary)	0.0757	0.101	0.0680	0.0775
Hourly wage (in $2022)	22.63	24.81	21.32	0.2032
Annual hours worked	1702.1	1665.5	1715.6	0.0916
Annual hours paid	1840.2	1804.7	1850.7	0.0595
Overtime hours paid	58.42	47.94	62.94	0.1199
Annual paid leave hours	138.0	139.2	135.1	0.0968
***Other fringe benefits***				
Paid vacation hours per year	68.73	68.64	67.73	0.0633
Paid national holiday hours per year	44.11	44.38	43.59	0.0678
Medical insurance offered (binary)	0.681	0.686	0.676	0.0325
Prescription drug insurance offered (binary)	0.668	0.673	0.663	0.0325
Dental insurance offered (binary)	0.418	0.475	0.395	0.1521
Life insurance offered (binary)	0.560	0.536	0.568	0.0014
Short-term disability offered (binary)	0.379	0.440	0.365	0.0494
Long-term disability offered (binary)	0.325	0.310	0.328	0.0323
Family leave offered (binary)	0.144	0.127	0.134	0.0441
Fixed paid sick time (binary)	0.369	0.396	0.324	0.1460
Consolidated sick plan PTO (binary)	0.221	0.181	0.228	0.0351
Health insurance cost per hour	2.393	2.588	2.271	0.1531
Non-production cost per hour	0.654	0.640	0.635	0.0355
***Main occupations (by share)***				
Office and administrative	0.162	0.170	0.160	0.0158
Sales and related	0.113	0.110	0.114	0.0303
Food preparation and serving	0.105	0.116	0.103	0.0216
Transportation and material	0.0829	0.0778	0.0856	0.0128
Production	0.0827	0.0674	0.0907	0.0984
Health practitioners and technicians	0.0614	0.0566	0.0628	0.0275
Installation, maintenance, and repair	0.0453	0.0385	0.0483	0.0386
Management	0.0432	0.0477	0.0405	0.0526
***Main industries (by share)***				
Healthcare and social assistance	0.163	0.158	0.162	0.0323
Retail trade	0.140	0.141	0.141	0.0066
Manufacturing	0.116	0.107	0.121	0.0792
Accommodation and food services	0.115	0.121	0.115	0.0195
Admin, support, waste management; remed. services	0.0668	0.0633	0.0676	0.0015
Professional, scientific, and technical services	0.0685	0.0804	0.0626	0.0351
Finance and insurance	0.0506	0.0483	0.0512	0.0081
Construction	0.0514	0.0489	0.0530	0.0240
Wholesale trade	0.0460	0.0478	0.0458	0
Transportation and warehousing	0.0433	0.0374	0.0453	0.0066
Firm size	623.0	664.0	584.1	0.0903
Observations	4,43,740	92,403	3,15,079	

Notes: NCS data from 2009 to 2022 (Bureau of Labor Statistics 2023c). Data are yearly and at the firm-job level; they are weighted by BLS provided weights. Minimum and maximum values not available due to data confidentiality reasons. According to BLS’ definition, “medical insurance” is health insurance without drug coverage. The “normalized difference” is calculated according to Imbens and Wooldridge (2009) where a value above 0.25 indicates covariate imbalance.

### 3.2. Main Outcome Variables

This paper evaluates how sick pay mandates affect firm propensities to offer mandated and non-mandated benefits, employee use of paid and unpaid sick leave, and firm costs directly related to sick leave. Our first outcome measures firm provision of paid sick leave as of March in a given calendar year. *Sick leave offered* is 1 if a job provides paid sick leave and 0 otherwise. Over all jobs and years, the average coverage rate is 63% (Table 1).

Our second outcome measures *paid sick hours taken* in the previous 12 months; if the specific job is filled by more than one employee, human resources administrators report average use among all employees in this job. Again, note that we do not observe the specific weekdays or calendar months of use. The sample average is 16.8 hours, which corresponds to taking just over two full workdays of paid sick leave.

Our third outcome measures *unpaid sick hours taken* in the previous 12 months. Unpaid sick leave may be a substitute for paid sick leave; recall that many employees (who are not covered by FMLA) gained access to unpaid sick leave through the mandates. The average unpaid sick days taken is 0.15 per employee and year.

Our fourth outcome measures *sick leave costs per hour worked* and is calculated by the BLS. The average is 27.5 cents per hour worked. Following the flow measure concept of sick leave utilization, it refers to the past 12 months before the first quarter interview. The BLS NCS survey administrators generate this variable and use employees’ own wage and own hours worked per year in the calculation. The variable assumes that sick hours represent 100% lost labor and does not consider changes in employee on-the-job productivity because of sick pay, or compensatory behavior by employees after returning to work.

The second panel of Table 1 lists job characteristics, that is, control variables and variables to stratify the sample in order to investigate effect heterogeneity. In particular, they measure full-time work (74%), unionization (8%), the hourly wage ($22.63), annuals hours worked (1,702), and annual hours paid (1,840) as well as paid overtime hours (58). Further, we know occupation and industry of each job. The three most common occupations are “office and administrative,” “sales,” and “food preparation and serving.” The three most common industries are “healthcare and social assistance,” “retail and trade,” and “manufacturing.”

### 3.3. Other Fringe Benefits

On average, American jobs offer 69 paid vacation hours and 44 paid national holiday hours per year. Moreover, 68% of all jobs offer health insurance^14^ and 56% offer life insurance. Short-term disability insurance is offered in 38% of all jobs and long-term disability insurance in 32% of all jobs (cf. Pichler and Ziebarth 2024). Fourteen percent of all U.S. jobs offer paid family leave.

## 4. Empirical Approach

Our objective is to estimate the effect of state-level sick pay mandates on provision of paid sick leave, use of paid and unpaid sick leave, labor costs, and non-mandated benefits. We use DD methods; our target parameter is the average treatment effect on the treated (ATT). States have adopted paid sick leave mandates at different points in time; thus, we use the methods proposed by Callaway and Sant’Anna (2021) (CS). The CS DD estimator is robust to bias from both forbidden comparisons (i.e., comparing later treated units to earlier treated units) attributable to dynamics in treatment effects as well as heterogeneity in treatment effects across treated units.

The central assumption of DD methods is common trends between adopting and non-adopting units. While this assumption is untestable as counterfactual outcomes are not observed, we follow the literature and estimate event studies to provide suggestive evidence on pre-mandate trends. Section 4.1 describes, first, the DD methods. Section 4.2 describes, second, the event studies.

### 4.1. Difference-in-Differences

Equation (1) outlines our DD specification as follows:


(1) \begin{eqnarray*}
y_{f,j,s,t}= \gamma _{s} + \delta _t + \phi D_{f}\times T_{s,t} + \mu _{f,j,s,t},
\end{eqnarray*}

where $y_{f,j,s,t}$ is one of the outcome variables (e.g., *paid sick leave offered*) at firm *f* in job *j* in state *s* and year *t*. $\gamma _{s}$ are state fixed effects and $\delta _t$ are year fixed effects. In additional specifications, we control for state paid time off (PTO) mandates and job-level covariates (part-time vs. full-time, and union vs. non-union).^15^

$D_{f}$ is an firm-specific treatment indicator, coded one for firms that have to comply with the mandates (considering mandate-specific size thresholds).^16^ These firms are located within states that implemented a sick pay mandate between 2009 and 2022.^17^ The interaction of $D_{f}$ with the vector $T_{s,t}$, where *s* refers to the state specific treatment timing, yields the binary DD variable. The interaction term is one for firms above the size threshold in states and time periods in which a paid sick leave mandate is in effect (see Online Appendix Table A.1, column 3).

The standard errors ($\mu _{f,j,s,t}$) are clustered at the state-level (Bertrand, Duflo, and Sendhil 2004). We use the doubly robust DD estimator proposed by Sant’Anna and Zhao (2020) that is based on stabilized inverse probability weighting and OLS. We use never treated units as our comparison group, but show robustness checks using “not yet treated.”

### 4.2. Event Study

We estimate and plot event studies to complement the DD method described above. To this end, we decompose the binary $T_{s,t}$ time indicator in equation (1) into a series of leads and lags around the effective date of each mandate using Callaway and Sant’Anna (2021). We report indicators for five or more years through one year in advance of the state-level mandates (“leads”, $\sum _{i=-5}^{-1}{Lead}_{f,i}$), the effective year of the mandate, and one through five or more years following the mandate (‘lags’, $\sum _{k=0}^{5}Lag_{f,k}$). We assign all states without a mandate a zero for all lead and lag variables. Our event study equation is as follows:


(2) \begin{eqnarray*}
y_{f,j,t} = \gamma _{f,j} + \delta _t + \kappa _{j} \sum _{i=-5}^{-1}Lead_{f,i} + \gamma _{k} \sum _{k=0}^{5}Lag_{f,k} + \rho X_{f,j,t} + \epsilon _{f,j,t}.
\end{eqnarray*}

Event studies offer two important extensions to the traditional DD model. First, visual examination of the normalized pre-mandate trends (i.e., the coefficient estimates on the lead indicator variables) allows us to test for the plausibility of the common time trends assumption. Second, inclusion of the lag variables allow treatment effects to vary over time in the post-mandate years. For example, if firms are slow to comply with the mandated benefits, allowing for dynamic treatment effects may be crucial. We note again that employees must learn about their rights, earn, and accrue sick time before they can claim sick pay; this suggests that effects may emerge over time.

### 4.3. Identification

Overall, we evaluate the average impact of the mandates adopted at the state-level between March 2009 and March 2022. If mandates are a reaction to pre-existing trends in the outcome variables in the treated states, we would identify such an endogenous implementation via our event study (i.e., coefficient estimates on the mandate lead variables that are statistically different from zero). Similarly, event studies provide evidence for anticipation effects.

The main remaining identification assumption is the absence of other confounding effects that are correlated with the staggered implementation of the mandates. Specifically, the implementation of the mandates and the outcome variables must not be correlated with a systematic, third, unobservable driving force. Note that the mandates were implemented at different times of the year, in January as well as in July (Online Appendix Table A.1), which adds to the credibility of the identifying assumption. Because we rely on variation over across 13 U.S. states from 2009 to 2022, compared to the canonical DD setting with just one treatment and one comparison group, other policies (or unobservables) contemporaneous to the treatments in all states inflicting a systematic bias are unlikely.

If the identification assumptions hold, equations (1) and (2) estimate internally valid causal mandate effects. The extent to which these estimates are externally valid for other U.S. states is difficult to assess. For such predictions, using estimates of regions whose labor markets are most similar to those in the state of interest is a promising approach. Our detailed heterogeneity analysis by industry, occupation, and both type of employee and employer will provide additional guidance.

## 5. Results

We begin this section by estimating equation (2). That is, we estimate event studies to elicit ITT effects of the state-level mandates on a range of outcomes. We then supplement these event studies with average DD post-reform estimates as in equation (1). As discussed, we routinely use the Callaway and Sant’Anna (2021) estimator that corrects for biases due to effect heterogeneity and dynamic treatment effects. Event time is unbalanced due to the staggered design; however, three states (CA, MA, and OR) include full event time observations ($\sum _{i=-5}^{-2}{Lead}_{f,i}$ to $\sum _{k=0}^{5}{Lag}_{f,k}$).

### 5.1. Impact of Mandates on Firms’ Benefit Provision and Employee Take-Up

**Figure 2.**
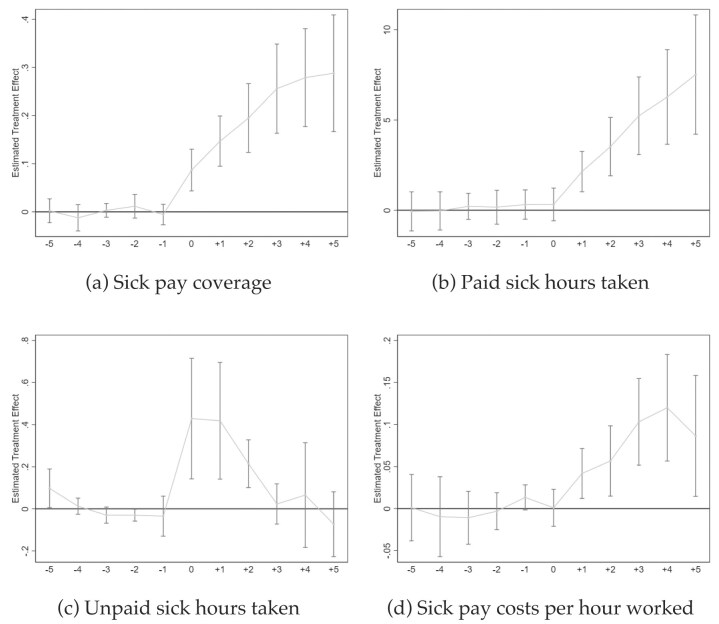
Event studies on main outcomes. Source: NCS data from 2009 to 2022 (Bureau of Labor Statistics 2023c). All graphs show Callaway and Sant’Anna (2021) event studies. Standard errors are clustered at the state level and the gray bars depict 90% confidence intervals. Event studies include year and state fixed effects. For more information about the sick pay reforms, see Online Appendix Table A.1.

*Event Studies: Main Outcomes*. Figure 2 plots events studies for our four main outcome variables. The *x*-axis of Figure 2 shows the normalized time dimension for all treatment states. The *y*-axis shows the treatment effect in natural units.

By examining the mandate leads, event studies allow us to asses the main identification assumption, that is, common trends. As seen, there are no differential trends between the treatment and comparison groups; the pre-mandate coefficient estimates are small in magnitude and the gray confidence bands entirely cover the zero line on the y-axis.

*Coverage*. Figure 2(a) documents a substantial increase of jobs with sick pay in the year of the mandate’s adoption. For example, in California, where the law became effective July 1, 2015, $\gamma =0$ refers to the first post-mandate year and March 2016. In the next three post-mandate years, $\gamma _{k} \sum _{k=1}^{3}{Lag}_{s,k}$, coverage rates further strongly increase to approach 30 ppt, and then flatten through $\gamma =5$. All post-mandate treatment effects are highly significant at conventional statistical levels.^18^

*Take-up*. Figure 2(b) and (c) show the dynamic effects on paid and unpaid sick leave use. Recall that the mandates grant employees access to unpaid *and* paid sick leave. After the mandates’ implementation, paid sick leave use strongly increases—linearly through $\gamma =5$. The linear increase in paid sick leave use is plausible as employees earn and accumulate sick leave credit over time.^19^

Regarding unpaid sick leave use in Figure 2(c), we observe nonlinear dynamic effects featuring an inverse U-shape. After employees in small firms gained the right to take unpaid sick days because of the mandates, we observe increases in use for the first three post-mandate data points, $\gamma _{k} \sum _{k=0}^{2}{Lag}_{s,k}$. Then, take up of unpaid sick hours starts to decline again and reverts back to the zero line in $\gamma =3$. This nonlinear effect is plausibly a function of how the sick pay mandates are designed—employees must first earn paid sick time credit through work. Hence, initially, employees (have to) take unpaid sick time. Once they have accrued sufficient paid sick time, employees increasingly take paid sick leave—and unpaid sick leave use decreases again. This nonlinear pattern suggests no significant medium to long-term effect on unpaid sick leave use.

*Labor Costs*. Finally, Figure 2(d) shows the event study for sick leave costs per hour worked. Again, there is no significant trending in pre-mandate years. Once employees begin to take paid sick time after being able to earn credit, labor costs increase. This is expected as sick leave costs are simply the product of paid sick hours taken and employees’ hourly wage.

*DD Models: Main Outcomes*. Table 2 reports the results from equation (1) for our main outcome variables. Each panel shows eight separate DD models. Panel A includes year and state fixed effects, whereas Panel B adds employee controls as well as control for PTO laws. Overall, the results are robust. For each of the four main outcomes and each panel, we report the findings from two regressions. In uneven columns, we report estimates from standard two-way fixed effects DD models that suffer potentially from bias but allow us to plot year fixed effects for the COVID-19 years 2020, 2021, and 2022. This approach pinpoints general underlying trends during the pandemic in a succinct manner within our standard framework.^20^ In even columns, we report Callaway and Sant’Anna (2021) DD estimates, which capture the average post-mandate effect; see Figure 2.

**Table 2.** Effect of mandates on coverage, utilization, and labor costs.

	Sick leave offered		Paid sick hours taken		Unpaid sick hours taken		Sick leave costs per hour	
Outcome	(1)	(2)	(3)	(4)	(5)	(6)	(7)	(8)
*Pretreatment mean*:								
*(in treated localities)*	0.6262		17.9862		0.1354		0.3378	
**Panel A**								
Sick leave mandate	0.189***	0.202***	4.472***	3.906***	0.180***	0.172**	0.085***	0.062***
($D_{c}\times T_t$)	(0.026)	(0.047)	(0.574)	(1.046)	(0.039)	(0.068)	(0.013)	(0.022)
Year 2020	0.079***		2.812***		−0.214***		0.066***	
	(0.014)		(0.659)		(0.073)		(0.013)	
Year 2021	0.085***		3.091***		−0.225***		0.058***	
	(0.014)		(0.711)		(0.075)		(0.012)	
Year 2022	0.087***		2.982***		−0.217***		0.044***	
	(0.014)		(0.625)		(0.077)		(0.010)	
Year FE	X	X	X	X	X	X	X	X
State FE	X	X	X	X	X	X	X	X
**Panel B**								
Sick leave mandate	0.187***	0.198***	4.367***	3.630***	0.187***	0.182**	0.084***	0.058***
($D_{c}\times T_t$)	(0.020)	(0.029)	(0.460)	(0.622)	(0.040)	(0.073)	(0.011)	(0.017)
Year FE	X	X	X	X	X	X	X	X
State FE	X	X	X	X	X	X	X	X
Employee controls	X	X	X	X	X	X	X	X

Notes: NCS data from 2009 to 2022 (Bureau of Labor Statistics 2023c). FE = fixed effects. Each uneven column in each panel stands for one Two-Way-Fixed Effects DD model as in equation (1); each even column stands for one Callaway and Sant’Anna (2021) model accounting for possible biases due to treatment dynamics and heterogeneity; ***, **, and * = statistically different from zero at the 1%, 5%, and 10% level. All models are weighted using NCS weights provided by the BLS. Employee controls: unionized job, part-time employment. Further, Panel B controls for PTO mandates. Standard errors clustered at the state level and reported in parentheses. All models have 4,43,740 firm-job observations. Firms below the firm size cutoff are coded as zero. See Online Appendix Table B.6 for results after dropping these observations.

*Coverage*. Column (2) of Panels A and B in Table 2 show that, on average, state-level sick pay mandates increase coverage rates, highly significantly, by 20 ppt over all post-mandate years. Relative to the pre-mandate coverage rates in treated states of 63%, the effects translate into an increase of 32%. Further, on average, during each COVID-19 year, paid sick leave coverage increases by 8 ppt throughout the United States. The effect is a weighted average of COVID-19 related emergency sick leave provisions and general time trends that were likely also driven by COVID-19 experiences with infections at the workplace; see A Better Balance (2021).

*Take-Up*. Columns (3)–(6) of Table 2 show the estimated take-up effects on paid and unpaid sick leave use in the 12 months prior to the March interviews. Column (4) shows robust evidence that paid sick leave use increases by almost 4 hours per year, which corresponds to a 22% increase relative to the pre-treament baseline. Scaling the increase in Panel A by the 20 ppt increase in coverage yields a 19 hours increase for marginal firms, or 2.4 paid sick days taken per year and newly covered employee. Like coverage, take-up is consistently higher during the pandemic with about three additional hours of paid leave taken in 2020, 2021, and 2022, compared to pre-COVID-19 years.

In column (6), the use of unpaid sick hours more than doubles to 0.17, with a scaled effect of 0.85 hours per marginal job. However, recall the nonlinear effect in Figure 2(c), suggesting no longer term impact on unpaid sick leave use. Interestingly, but potentially expected, during the pandemic unpaid sick leave use is significantly below pre-COVID-19 levels and a statistically significant −0.22 for each of the three years of the pandemic.

*Labor Costs*. Column (8) shows effects for labor costs per hour worked. Labor costs are important to assess in this context because mandate critics commonly cite rising labor costs and depressed labor demand as reasons against government mandated sick pay (Kruth 2018). We find that mandates increase sick leave costs per hour worked by 6.2 cents (column (8), Panel A). Scaling this cost increase by the 20 ppt increase in coverage rates, costs increase by 31 cents per hour for the marginal employer.

We note that this cost estimate is a static calculation. In particular, the calculation does not consider possible changes in work productivity attributable to the mandate. For instance, overall work productivity could increase because employees can recover from their sickness, work moral among employees could increase, or employees may (over-) compensate for lost labor after their sick leave. On the other hand, shirking and a lower work morale among employees who are not on sick leave (and therefore must cover for their sick coworkers) could reduce productivity.

However, the labor cost estimate implicitly considers potentially lower infection rates at the workplace and thus a reduced need for sick leave (cf. Pichler and Ziebarth 2017; Stearns and White 2018; Pichler, Wen, and Ziebarth 2020). If total sick hours taken decreases in some firms or occupations as a result of less presenteeism behavior and fewer infections, our labor cost estimate would implicitly consider such an effect.

#### Effect Heterogeneity.

 Next, we explore effect heterogeneity by type of job and by type of firm. Given the large inequalities across jobs in the pre-mandate era, one would hypothesize that heterogeneity in mandate effects should be large as well. In other words, we expect the mandates to have more bite in part-time and low-wage jobs where voluntary coverage is low(er) in pre-mandate years. Partly, this hypothesis is mechanically true due to “ceiling effects:” For jobs with very high pre-mandate coverage, the potential increase in coverage to a maximum of 100% (of all jobs) has, by construction, a lower ceiling than for jobs with very low pre-mandate coverage rates. Analogously, we hypothesize take-up and labor cost effects to be large in jobs with low pre-mandate coverage.

*Type of Firm*. To this end, we re-estimate equation (1) on split samples, for example, full time versus part-time jobs in Panel A of Table 3. As seen, our main hypothesis is on target. Coverage rates increase by 13 ppt in full-time jobs, on average, but by 38 ppt—three times as much—in part-time jobs. Average absolute take up increases by 3.3 hours (full-time) and 4.2 hours (part-time). However, the scaled effect is larger for full-time (25.8 hours) as compared to part-time (11.2 hours) jobs. The reason is accrual rates that are identical for both types of jobs: Obviously, part-time workers accrue fewer credit and are thus unable to take sick time at the same rate as full-time workers. Also, to the extent that they do not work every day, part-time workers have fewer possibilities to fall sick on a given day during the year.

**Table 3.** Effect heterogeneity of mandates: by firm and type of job.

Outcome	Sick leave offered (1)	Paid sick hours taken (2)	Unpaid sick hours taken (3)	Sick leave costs per hour (4)
**Panel A: Full-time vs. part-time**				
Full-time	0.128***	3.313***	0.071	0.046*
	(0.033)	(1.124)	(0.073)	(0.028)
*Pretreatment mean*:	0.7789	23.5940	0.1468	0.4327
*(in treated localities)*				
Part-time	0.375***	4.196***	0.456***	0.080***
	(0.040)	(0.422)	(0.118)	(0.019)
*Pretreatment mean*:	0.2267	3.3119	0.1054	0.0895
*(in treated localities)*				
**Panel B: Union vs. non-union**				
Union	0.035	0.491	−0.331	−0.056
	(0.038)	(2.385)	(0.283)	(0.126)
*Pretreatment mean*:	0.7523	26.5441	0.4262	0.6562
*(in treated localities)*				
Non-Union	0.219***	4.293***	0.226***	0.077***
	(0.058)	(0.993)	(0.085)	(0.016)
*Pretreatment mean*:	0.6121	17.0272	0.1028	0.3021
*(in treated localities)*				
**Panel C: Large vs small employers**				
Big firm ($> $500 employees)	0.059*	0.318	−0.224	−0.035
	(0.032)	(1.812)	(0.152)	(0.067)
*Pretreatment mean*:	0.8469	29.9609	0.1771	0.7022
*(in treated localities)*				
Small firm ($< $50 employees)	0.339***	5.926***	0.405***	0.090***
	(0.043)	(0.611)	(0.144)	(0.017)
*Pretreatment mean*:	0.4844	11.7839	0.1584	0.1766
*(in treated localities)*				
**Panel D: Higher vs. lower educated employees**				
Share with college $\ge$ median	0.190***	3.198**	0.188*	0.058
	(0.061)	(1.625)	(0.100)	(0.039)
*Pretreatment mean*:	0.6349	19.4501	0.0837	0.4634
*(in treated localities)*				
Share with college $< $ median	0.214***	4.393***	0.149*	0.046***
	(0.024)	(0.646)	(0.088)	(0.009)
*Pretreatment mean*:	0.6349	19.4501	0.0837	0.4634
*(in treated localities)*				

Notes: NCS data from 2009 to 2022 (Bureau of Labor Statistics 2023c). Each cell stands for one Callaway and Sant’Anna (2021) model accounting for possible biases due to treatment dynamics and heterogeneity; ***, **, and * = statistically different from zero at the 1%, 5%, and 10% level. All models are weighted using NCS weights provided by the BLS. Standard errors clustered at the state level and reported in parentheses. All models control for year and state fixed effects (FE). For event studies, please see Online Appendix Tables 5 and 6.

Nevertheless, our conjectures are on target for unionized versus non-unionized jobs (Panel B) as well as firms with less than 50 versus 500 or more workers (Panel C). Here, we observe much larger percentage point increases in non-unionized compared to unionized jobs, not only for coverage (21.9 vs. 3.5 ppt), but also for paid hourly use (4.3 vs. 0.5), unpaid hourly use (0.2 vs. −0.3) and labor costs (0.08 vs. −0.06). We also observe much larger coverage increases of 33.9 ppt for small firms with low pre-mandate coverage of just 48% vs. 85% for large firms with coverage increases of 5.9 ppt due to the mandates. Take-up of paid sick leave increases by a highly significant 5.9 hours for small firms and labor costs by a significant 9 cents per hour worked (paid use and labor cost increases are not significant for larger firms). Take-up of unpaid leave is also larger for small firms (0.4 vs. −0.2).

*Type of Job*. To test whether job-specific human capital and the substitutability of workers matter, we also stratify jobs by the share of college graduates. To do so, we create 516 industry-occupation cells in the representative American Community Survey, and differentiate these cells by whether they have an above or below average share of employees with college degrees (IPUMS USA 2024).^21^ Then, we split the sample based on this variable. However, Panel D of Table 3 shows effect sizes that are similar to the sample average.

*Industry and Occupation*. Next, we investigate effect heterogeneity by industry and occupation. The detailed results are in Online Appendix Table B.1. Again, our main conjectures are confirmed. Mandates have most bite in industries and occupations with very low rates of sick leave provision pre-mandate, such as in “construction” (40%), “administration, support and waste management” (37%), “accommodation and food services” (18%), or “food preparation and serving” (18%). In addition to disproportional increases in access, these industries and occupations see increases in paid sick leave use of between 4 and 8 hours per year (unscaled), and also significant increases in labor costs of between 4.4 and 13.6 cents per hour worked (also unscaled).

**Figure 3.**
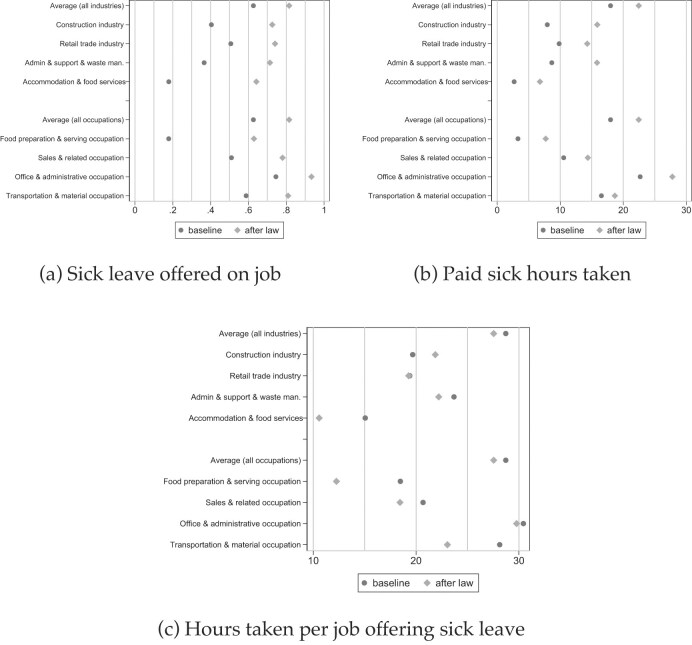
Coverage effect heterogeneity by industry and occupation (I). Source: NCS data from 2009 to 2022 (Bureau of Labor Statistics 2023c). Results with additional outcomes are in Online Appendix Table B.1; event studies are in Figure 7. Industries and occupations are sorted by the weighted frequency of the biggest industries and occupations. That is why the average of the industries and occupations shown does not equal the sample average; please see Table 1 for the full list of industries and occupations. Figure (c) reports the ratio of (b) and (a).

Figure 3 graphically illustrates the effect heterogeneity in access by industry and occupation. The dark dots report the baseline coverage rates, whereas the lighter diamonds show the post-mandate coverage rates (i.e., the summation of the baseline rates and treatment effects in Online Appendix  Table B.1). Pre-mandate, there was substantial inequality in access. The mandates have substantially reduced this inequaliy; jobs with low pre-mandate coverage rates experience much larger increases (Figure 3a). Paid sick leave use has increased across all industries and occupations, but to a different extent, which is certainly a function of the type of job, worker composition, pre-mandate access, and leave taking behavior. For example, Figure 3(c) shows paid sick time use scaled by coverage levels in Figure 3(a), that is, the figure shows sick time use *per job that offers the benefit*. For almost all industries and occupations, sick leave use per job has *decreased* in the first post-mandate years, as newly covered workers tend to have lower-than-average use, simply because they have less sick time credit in their accounts.

One exception is the construction industry where the use of paid sick hours per job has increased from 20 to 22 hours per year. We see this pattern for the construction industry although a large share (of more than 20 ppt) of construction jobs gain coverage because of the mandates. The changes in sick leave use (per job with sick leave) may either be explained by the time it takes to accrue sick time for workers who just gained the right to earn sick time (Cronin, Harris, and Ziebarth 2022), and/or by differences in labor supply elasticities, which may be a function of pre-mandate sick leave behavior, mental or physical job strain, labor composition, and how infections at the workplace change when more employees take sick leave (cf. Pichler, Wen, and Ziebarth 2021; Andersen et al. 2023).

*Inequality Within Firms*. While the discussion above has shown that the mandates have reduced inequality in sick pay access across types of jobs and firms, by industry and occupation, it remains unclear whether inequality across jobs *within* firms has also decreased. Online Appendix Figure B.1 uses solely states and years without a mandate in place; it then plots a bar diagram showing the fraction of jobs within firms that come with voluntary sick pay. We observe heaping at both 0 and 1 representing firms that offer no sick pay at all, or sick pay for all jobs. Nevertheless, clearly a large share of jobs only offer sick pay in some of their jobs, represented by the fractions that are roughly equally distributed between 0.2 and 0.9.

**Figure 4.**
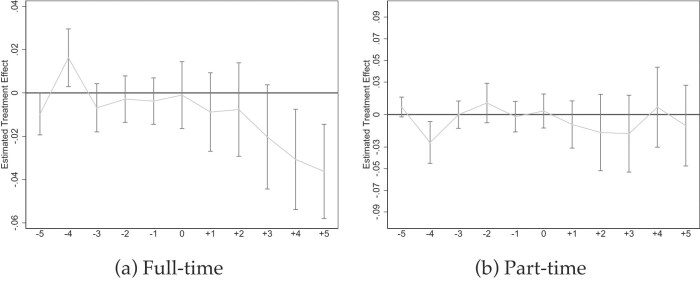
Effects on inequality of provision of paid sick leave within firm. NCS data from 2009 to 2022 (Bureau of Labor Statistics 2023c). All graphs show Callaway and Sant’Anna (2021) event studies where the outcome is whether sick pay is provided for some but not all full-time (a) or part-time (b) jobs in the firm. Standard errors are clustered at the state level and the gray bars depict 90% confidence intervals. Event studies include year and state fixed effects. For more information about the sick pay reforms, see Online Appendix Table A.1.

To test whether the mandates reduced such inequality within firms, we generate two outcome variables and use them in standard event study models as in equation (2): The first, Figure 4(a), indicates whether sick pay is provided for some but not all full-time jobs in the firm. Figure 4(b) does the same for part-time jobs. We see a significant decrease in coverage inequality among full-time jobs within the same firm. The decrease increases linearly over the post-mandate periods and becomes significant in $\gamma =+4$. In $\gamma =+5$, inequality approaches 0.04 off a baseline of 0.13, implying a decrease of 31%.

On the other hand, we observe no such pattern for part-time jobs in Figure 4(b), but we also cannot exclude relative large effect sizes of [0.5; 0.5]. A potential explanation is the exemptions that many mandates entail. For example, Connecticut explicitly only covers full-time employees and the mandates in Maryland, Minnesota, and Vermont specifically have “hours per week worked” requirements. Further, other mandates, for example, in Massachusetts and Oregon, exempt small firms that have larger shares of part-time workers.

**Figure 5.**
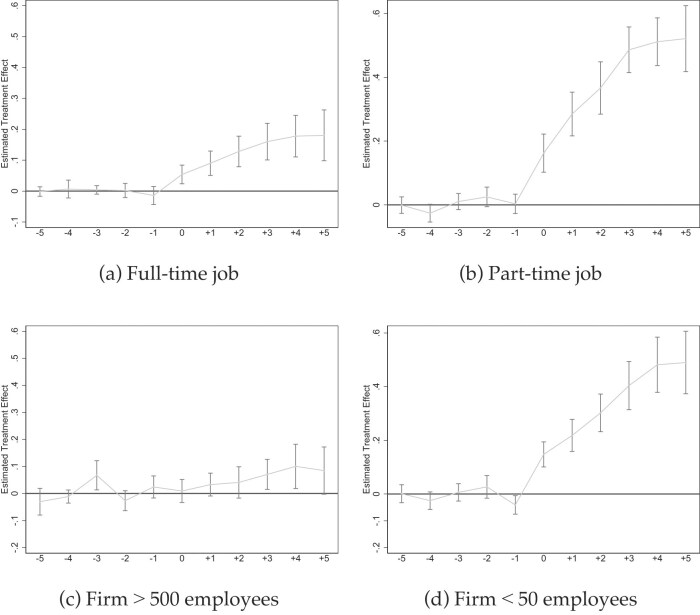
Event studies by type of job (I). Source: NCS data from 2009 to 2022 (Bureau of Labor Statistics 2023c). All graphs show Callaway and Sant’Anna (2021) event studies by type of job as indicated. When mandates exempt small firms, they are coded as such or dropped (see Online Appendix Table B.6). Standard errors are clustered at the state level and the gray bars depict 90% confidence intervals. Event studies include year and state fixed effects. For more information about the sick pay reforms, see Online Appendix Table A.1.

**Figure 6.**
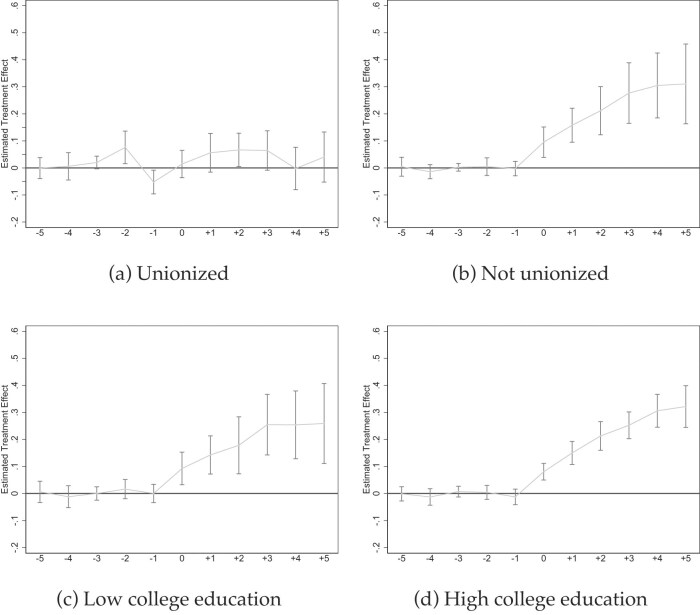
Event studies: by type of job (II). Source: NCS data from 2009 to 2022 (Bureau of Labor Statistics 2023c). All graphs show Callaway and Sant’Anna (2021) event studies by type of job as indicated. In subfigures (c) and (d), the job is flagged according whether it has a below or above share of college educated employees. For this analysis, we rely on to 516 occupation-industry cells in the 2010 American Community Survey; those cell have identical classifications in the NCS. Standard errors are clustered at the state level and the gray bars depict 90% confidence intervals. Event studies include year and state fixed effects. For more information about the sick pay reforms, see Online Appendix Table A.1.

**Figure 7.**
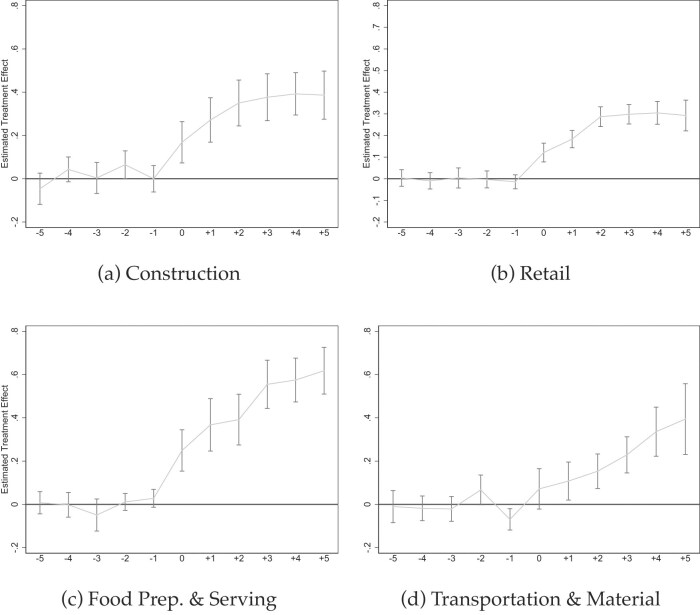
Event studies: Select industries and occupations. NCS data from 2009 to 2022 (Bureau of Labor Statistics 2023c). All graphs show Callaway and Sant’Anna (2021) event studies by industries (a and b) and occupations (c and d) as indicated. Standard errors are clustered at the state level and the gray bars depict 90% confidence intervals. Event studies include year and state fixed effects. For more information about the sick pay reforms, see Online Appendix Table A.1.

*Event Studies Illustrating Coverage Heterogeneity*. Figures 5–7 show event studies mirroring what the point estimates in Table 3 and Online Appendix Table B.1 reveal: much steeper slopes for jobs with lower pre-mandate baseline coverage rates. The medium-term increases for part-time jobs (Figures 5b), small firms (Figures 5d), and occupations such as “food preparation and serving” (Figure 7c) or “transportation” (Figure 7(d) approach 50 ppt in $\gamma =5$ but visibly flatten over time. The long-term increases in sick pay coverage for non-unionized jobs approach 30 ppt (Figure 6b), and those for full-time jobs 20 ppt (Figure 5a). In contrast, the effects are below 10 ppt for large firms and unionized jobs (Figures 5c and  6a).

*Heterogeneity by State. *As our final analysis of mandate heterogeneity, we study coverage effects for select states. In the Online Appendix, we show raw time trends for California (Figure B.2a), Arizona (Figure B.2b), Oregon (Figure B.2c), and Connecticut (Figure B.2d), and then show event studies for the same states in Figure B.3.

In all states but Connecticut (where the mandate only covers 20% of the workforce), the event studies show what the raw trends forecast: relatively flat and common time trends pre-mandate, and then increasing coverage rates post-mandate. Coverage approaches 100% over time, but effects take 4–5 years to rise to this level, potentially due to mandate unawareness by firms or lags in reporting. In Connecticut, one extreme of the spectrum with a very lax mandate and many exemptions, one observes upward trends in coverage but no visible reform effect. In California, the other extreme with barely any exemptions, we observe the strongest mandate-driven increase in sick leave coverage of about 30 ppt. This effect is reached in the fourth March after the mandate’s inception, that is, in March of 2019. As a consequence, when COVID-19 hit one year later, sick leave coverage among workers in California was much more comprehensive than anywhere else in the U.S.

### 5.2. Effects on Hours Worked and Type of Sick Plan

**Figure 8.**
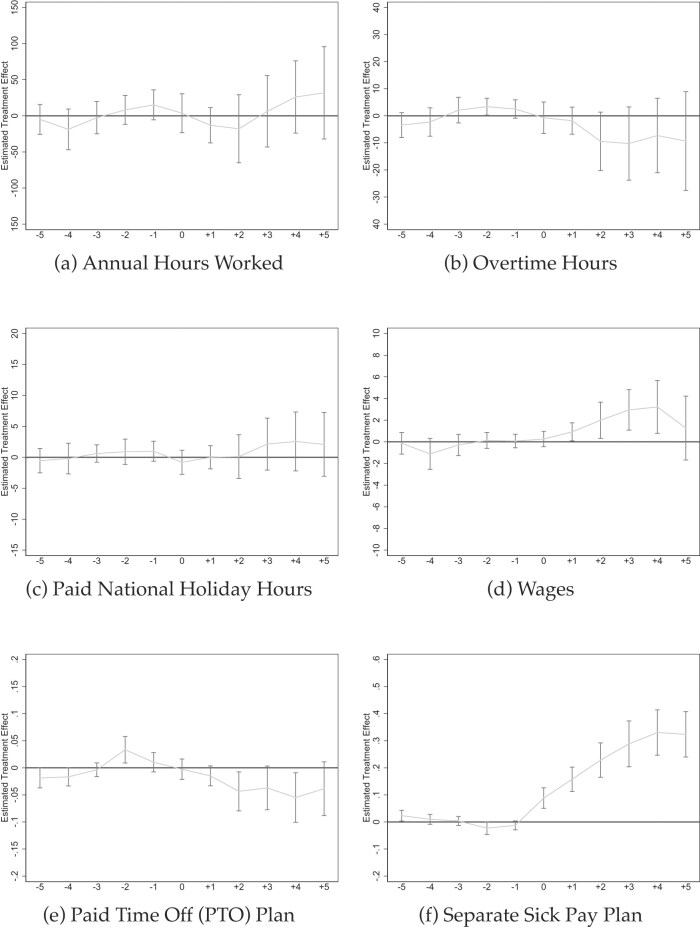
Event studies: effects on hours worked, overtime, wages. Source: NCS data from 2009 to 2022 (Bureau of Labor Statistics 2023c). All graphs show Callaway and Sant’Anna (2021) event studies. Standard errors are clustered at the state level and the gray bars depict 90% confidence intervals. Event studies include year and state fixed effects. For more information about the sick pay reforms, see Online Appendix Table A.1.

In Figure 8, we show effects on (a) annual hours worked, (b) overtime hours, (c) paid national holiday hours, and (d) wages. The post-mandate coefficient estimates are in Online Appendix Table B.2.

Although the effects are imprecisely estimated, we do not observe much evidence for structural changes in the number of annual hours worked; the coefficient estimate is 0.6% of the mean and not statistically significant. For overtime hours, we observe some more systematic downward trending; however, the coefficient estimate of −5.3 (hours per year) only becomes marginally significant when adding employee controls. One potential explanation could be that the need to work overtime decreases when employees accumulate earmarked time off to take care of sick children or to take doctor appointments. For national holiday hours, we do not find systematic changes, and the coefficient estimates in our standard models in Panels A and B of Online Appendix Table B.2 are non significant. As for hourly wages, there is some suggestive evidence for modestly rising wages but the effect appears to return to the baseline.^22^ In any case, we do not find any evidence for reduced wages as textbook models would suggest (Summers 1989; Gruber 1994). This finding is in line with prior research on the wage effects of sick pay mandates (Pichler and Ziebarth 2020).

However, the evidence regarding the type of sick plan that firms set up to comply with the mandates is very clear: Separate sick plans overwhelmingly drive almost the entire coverage effect (Figure 8f) as opposed to a “consolidated leave plan” (Figure 8c). The latter plans are also called consolidated “paid-time-off” (PTO) plans and have become increasingly popular in the United States. Under a PTO plan, employers do not provide a *separate* number of days for sick leave, vacation, or parental leave, but instead aggregate or *consolidate* the total number of paid leave days per year, independent of reason (Lindemann and Miller 2012). The BLS reports that the average consolidated PTO plan has accumulated 19 days of paid leave credit after 5 years of service with the employer (Bureau of Labor Statistics 2023d). Table 1 shows that 22% of all jobs offer a consolidated PTO plan. PTO plans are in compliance with sick pay mandates if they are at least as generous as the sick leave accounts required by the law (ADP 2016).

### 5.3. Effects on Non-Mandated Benefits: Job Upscaling

**Figure 9.**
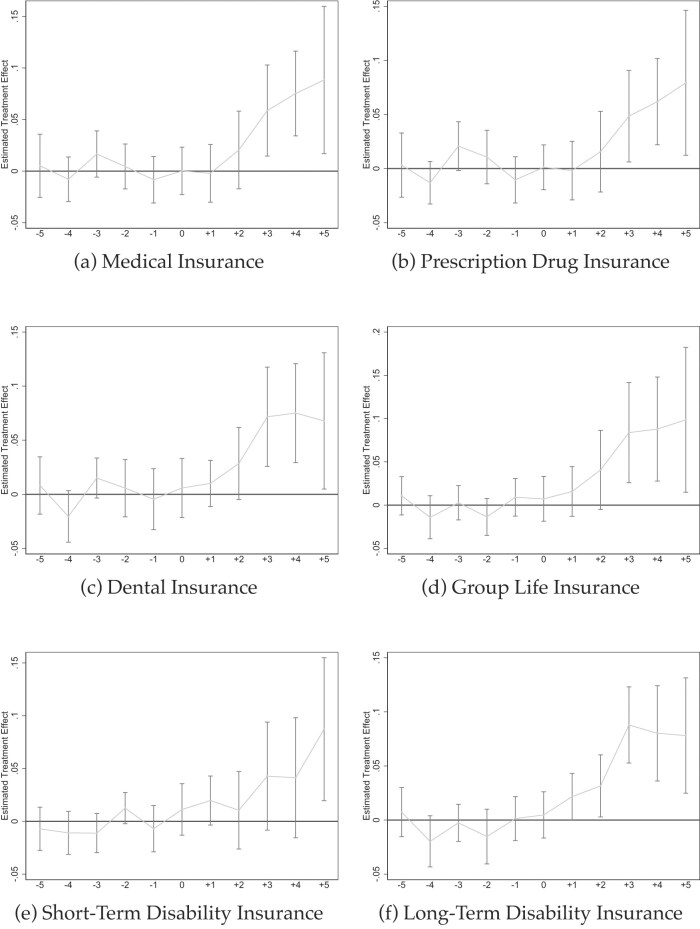
Event studies: effects on non-mandated benefits. Source: NCS data from 2009 to 2022 (Bureau of Labor Statistics 2023c). All graphs show Callaway and Sant’Anna (2021) event studies. Standard errors are clustered at the state level and the gray bars depict 90% confidence intervals. Event studies include year and state fixed effects. For more information about the sick pay reforms, see Online Appendix Table A.1.

Table 4 and Figure 9 report estimates on non-mandated benefits. Such fringe benefits are plausibly valuable to employees, but costly to firms and not mandated. Hence, one could hypothesize that firms curtail them to offset the increased sick leave costs, see above. We thus test for compensatory and spillover effects of mandating paid sick leave.

**Table 4.** Effect of sick leave mandates on non-mandated benefits.

	Insurance plans				Disability		Family	Paid sick leave	
	Health	Presc. drug	Dental	Group life	Short-term	Long-term	leave	Fixed	Consolidated
	(1)	(2)	(3)	(4)	(5)	(6)	(7)	(8)	(9)
*Pretreatment mean*:	0.6863	0.6729	0.4753	0.5365	0.4404	0.3101	0.1268	0.3965	0.1806
*(in treated localities)*									
Panel A									
Sick leave mandate	0.036*	0.029	0.037**	0.050**	0.034	0.047***	0.016	0.233***	−0.034**
	(0.019)	(0.018)	(0.017)	(0.025)	(0.021)	(0.016)	(0.011)	(0.038)	(0.015)
Year FE	X	X	X	X	X	X	X	X	X
State FE	X	X	X	X	X	X	X	X	X
Panel B									
Sick leave mandate	0.028**	0.022*	0.031**	0.045***	0.033**	0.041***	0.016*	0.230***	−0.034***
	(0.012)	(0.012)	(0.013)	(0.015)	(0.016)	(0.013)	(0.009)	(0.029)	(0.013)
Year FE	X	X	X	X	X	X	X	X	X
State FE	X	X	X	X	X	X	X	X	X
Employee controls	X	X	X	X	X	X	X	X	X

Notes: NCS data from 2009 to 2022 (Bureau of Labor Statistics 2023c). Presc. drug = prescription drug. FE = fixed effects. Each cell stands for one Callaway and Sant’Anna (2021) model accounting for possible biases due to treatment dynamics and heterogeneity; ***, **, and * = statistically different from zero at the 1%, 5%, and 10% level. All models are weighted using NCS weights provided by the BLS. Employee controls: unionized job, part-time employment. Further, Panel B controls for PTO mandates. Standard errors clustered at the state level and reported in parentheses. All models have 4,43,740 firm-job observations except for models (8) and (9), where we observe sick leave plans for 3,92,225 job year pairs. For event studies, please see Figure 9.

Figure 9 shows some potentially unexpected results. Contrary to expectations and textbook model predictions (Summers 1989), our event studies show relatively clear *positive* spillover effects of mandating paid sick leave on a range of fringe benefits such as medical, prescription drug, and dental insurance (Figure 9a–c), group life insurance (Figures 9d) as well as short- and long-term disability insurance (Figure 9e and f). The post-mandate coefficient estimates range from 3.2% for prescription drug coverage to 9.3% for life insurance and 15.1% for long-term disability insurance. In other words, we find clear evidence for *crowding-in* of non-mandated benefits.

We call this finding “job upscaling.” We explain job upscaling through increased provision of non-mandated fringe benefits as follows: Firms use it to attract skilled labor and signal high-quality jobs. This phenomenon represents an effort by (some) employers to differentiate themselves. Note, however, that job upscaling affects “only” 3–4 ppts of all jobs, that is, an overall small share of jobs. Firms that offer sick pay voluntarily significantly differ from firms that did not offer it pre-mandate^23^: They are bigger, more likely to be unionized, and have a higher share of full-time jobs that are also better paid. Importantly, they are about twice as likely to offer medical (83% vs. 43%), prescription drug (81% vs. 42%), and dental insurance (53% vs. 23%) and are more than twice as likely to provide group life (71% vs. 30%), short-term disability (47% vs. 22%), and long-term disability (44% vs. 13%) insurance.

How employers qualitatively assess the effects of the mandates nicely illustrates how this job upscaling mechanism operates at the firm level; see page 20 of Boots, Martinson, and Danziger (2009):

“The policies I had in place before were there to reduce turnover and get better employees—and they did have an effect. But now, since the new ordinance, employees will have the same benefit no matter where they work.”

Or, by another employer:

“Now my part-time employees are getting to be equal to my full-timers, those full-timers are upset that they’re getting the same benefits—they feel mistreated. There needs to be some distinction for those that work full time and have been working for me for a while.”

In other words, prior to the mandates, firms used the voluntary provision of fringe benefits to attract qualified workers, signal “good jobs,” and differentiate job quality by type of work. After the mandate, as all jobs come now with sick pay, this differentiation through voluntary sick pay provision falls flat. This is why, apparently, some firms then decided to offer other non-mandated benefits such as short- and long-term disability insurance.

### 5.4. Robustness

Finally, we aggregate our data (a) at the firm-level (Online Appendix Table B.3), (b) at the county-level (Table B.4), as well as (c) at the state-level (Table B.5). Note that these aggregations create some imprecision as the sample of firms changes over time. Therefore, estimated effects might be due to actual treatment effects (changes within a firm over time) or changes in the firm composition over time. However, our results show that the estimated effects are fairly robust to these different types of aggregation. As observed for all outcomes and model specifications, the results with aggregate data are very similar to our results based on job-level data.

Further, in Online Appendix Tables B.6–B.9, we conduct additional falsification tests. For example, while we code firms below mandate thresholds as not treated in states that exempt small firm, Table B.6 excludes these observations from the sample. Further, while our sample is representative for the United States, the representativeness for smaller states might be limited. To see whether smaller states drive our results, we solely keep California and all untreated states in Table B.7. As seen, although the point estimates increase slightly (for coverage and take-up) when focusing on California, they are not appreciably different from our main results.

Table B.8 also replicates Table 2 but uses the “not yet treated” as controls, not the “never treated” as in our standard approach; see Callaway and Sant’Anna (2021). As seen, the results are robust. They are also robust to solely keeping treated states with full event time observations (CA, MA, and OR), see Figure B.4. Finally, Table B.9 drops the COVID-19 years 2020–2022 and shows robust findings.

## 6. Discussion and Conclusion

This paper evaluates how state-level sick pay mandates operate at the firm-job level in the United States. We leverage the experiences of 12 U.S. states with a total of about 50 million employees. Using NCS from 2009 to 2022, coupled with Callaway and Sant’Anna (2021) DD and event study methods, we exploit the policy-induced variation in the implementation of the mandates since 2012. The NCS is a BLS survey at the firm-job level specifically designed to measure wage and non-wage compensation.

Our findings address important gaps in the economics literature on labor market inequalities and employer mandates more broadly. The United States has one of the least generous paid leave systems among OECD countries (Adema, Clarke, and Frey 2016; Raub et al. 2018; OECD Policy Responses to Coronavirus 2020). Federal minimum standards concerning paid vacation, paid parental leave, paid eldercare, and paid sick leave are largely absent, leading to inequality in the voluntary provision of such benefits by firms and across jobs. In general, better paying full-time jobs for higher educated employees tend to offer paid leave benefits, whereas part-time and low-income jobs, especially in the service sector, do not. An important question is to what extent employer mandates are effective in providing and facilitating the provision and use of such benefits; or whether they have unintended consequences and lead to substantially higher labor costs, and a reduction of non-mandated benefits.

We find that mandates are highly effective in increasing on-the-job access to paid sick leave. Four to five years after the mandates’ implementation, coverage rates have increased by 30 ppt from a baseline of 63%. Heterogeneity in mandate bite is large. In general, industries, occupations, and jobs with low voluntary provision (of sick pay absent mandates) have experienced the largest increases in coverage as a result of the mandates. For example, fewer than half of all jobs in small firms as well as in the “construction” or “accommodation and food services” industry offer paid sick leave absent a mandate. Further, we find that mandates have more bite, the more comprehensive the mandate is, such as the mandate in California. In any case, mandates decrease inequality in sick pay access across occupations, industries, and type of jobs, but also within firms: The likelihood that firms offer paid sick leave to some, but not all, full-time jobs linearly and significantly decreases over time after the mandates’ implementation.

As expected, we also find a significant increases in take up of paid sick leave. Employees in newly covered jobs take, on average, two additional sick days per year in the first five post-mandate years. Utilization is linearly increasing over time, whereas sick pay costs flatten after 4 years into the mandate. Our findings also suggest that use of unpaid leave sick will not increase significantly in the long-run when employees will have accumulated enough paid sick time.

Finally, contrary to our initial priors, we find that mandates *increase* the likelihood that firms offer non-mandated fringe benefits such as short- or long-term disability policies. We dub that phenomenon “job upscaling.” This finding is akin to the positive wage spillovers of higher minimum wages to higher income jobs, as shown in Cengiz et al. (2019). Apparently, (some) firms see the need to signal high quality jobs and to attract skilled labor through the provision of non-mandated benefits. As one employer puts in in a post-mandate survey:

“The policies I had in place before were there to reduce turnover and get better employees—and they did have an effect. But now [...] employees will have the same benefit no matter where they work.” Boots, Martinson, and Danziger (p. 20, 2009)

As states continue to implement sick pay mandates, more empirical evidence on the indented and unintended consequences of these mandates will become available. We look forward to fruitful discussions among social scientists.

## Supplementary Material

jvaf008_Maclean_Pichler_Ziebarth_Online_Appendix

jvaf008_Maclean_Pichler_Ziebarth_Replication_Files

## Footnotes

1 Paid family and medical leave provisions have also been implemented and are under discussion; see Pichler and Ziebarth (2024) for a comparison. Family leave is similar to parental leave and primarily designed to provide paid leave before and after birth or adoption (Bana et al. 2023), whereas medical leave is equivalent to long-term sick leave in Europe (Ziebarth 2013).

2 One exception is Pichler and Ziebarth (2020), who rely on administrative Bureau of Labor Statistics data using the Quarterly Census of Employment and Wages. However, Pichler and Ziebarth (2020) solely study employment and wage effects *in the aggregate, at the county level*. Pichler and Ziebarth (2020) have an entirely different focus and uses synthetic control group methods to assess whether there is macro-level evidence that mandates could reduce employment or wage growth (a main argument against mandates). The paper does not find much evidence for that. In contrast, this paper uses micro-level data at the *firm-job level* and studies different outcomes. Ahn and Yelowitz (2015) use American Community Survey data to assess the labor market effects of the first state law in Connecticut. They find small and non-significant, positive labor force participation effects.

3 We omit D.C. from our analysis as D.C already implemented the mandate in 2008 and thus we only observe post-treatment years. Further, three states passed PTO mandates, which provide a minimum number of paid leave days, irrespective of the reason for leave taking.

4 Additional unpublished papers or papers outside of economics find that women are at a higher risk of working sick (“presenteeism”) and that mandating access improves their health (Susser and Ziebarth 2016; Slopen 2023). Further, studies exploiting variation in U.S. mandates find a higher employee productivity and firm profitability as well as reduced firm bankruptcy (Chunyu, Volpin, and Zhu 2022; Miller 2022). Ahn and Yelowitz (2016) exploit variation in the strength of the flu for identification, similar to Cronin, Harris, and Ziebarth (2022), and find that sick leave coverage increases the use by 1.2 days per year per work, comparable to this study.

5 Gilleskie (1998, 2010) represent notable exceptions on early U.S. sick leave research. Gilleskie (1998) uses 1987 data from the Medical Expenditure Survey to estimate structural parameters in a discrete choice dynamic optimization problem where individuals decide on missing work due to acute illness. Policy simulations on providing universal paid sick leave increases sick leave use and also outpatient care use, suggesting that health care and sick leave are substitutes. Gilleskie (2010) uses a similar approach and finds that men’s labor supply elasticity with respect to sick leave benefits is larger than women’s.

6 Sick leave differs from parental leave in both aim and scope. Parental leave is typically implemented with the objective of balancing employees’ family and work responsibilities and addressing gender inequality in the workplace.

7 In Online Appendix Table  A.1, all but one state (and D.C.) require at least 1 hour of sick leave credit per 30–40 hours worked. Vermont is an exception with 52 hours and Washington DC requires 43 hours.

8 An open question for future research is employee sick leave behavior when changing jobs. The current laws do not specify any mandated pay out rate. This incentivizes employees to take their accrued sick time prior to leaving the job.

9 Our findings are robust to including fully or partially treated counties. These results are available upon request.

10 Technically, the NCS is an establishment-level survey.

11 Note that the NCS is not representative at the state-level. To the best of our knowledge, no state-level representative dataset exists. If our identification assumptions hold, non-representativeness at the state level is no threat to the internal validity of our estimates.

12 Note that within a firm-job cell, there can be multiple employees. When there are multiple employees, the NCS reports the average value.

13 Thus, for all states, the first post-mandate year only partially records utilization, for example, for CA and MA, 9 months are counted (Online Appendix Table A.1).

14 To be precise, here, we use what the BLS labels “medical insurance.” This variable does not necessarily cover prescription drugs.

15 In previous versions of this paper, we also included firm-job fixed effects in some specifications. However, as the dataset is a short rotating panel where firms are only sampled up to 3 years, this approach heavily attenuates the treatment effect. The reason is that not all firms are observed before and after the treatment and even if they are, they feature at most two post-mandate years. For these specifications and also the Bacon Decomposition (Goodman-Bacon 2021), please see Maclean, Pichler, and Ziebarth (2024).

16 Note that firms below the threshold are included in the comparison group. In a robustness check, results available on request, we exclude small firms from the comparison group and find slightly larger effects (although 95% confidence intervals generally overlap, which makes us reluctant to overstate any heterogeneity).

17 In particular, treatment status is absorbing, that is no states repeal their paid sick leave mandate. Moreover, as mentioned earlier, in the main specification, we exclude all counties and cities, which passed county-level or city-level mandates. However, our findings are robust to including those treated counties in the sample. As mentioned in Section 2, one complication with the city-level mandates is that the city boundaries where the mandate applies rarely coincide with the county boundaries, which is why we elect to exclude the entire county from the analysis.

18 Similar to non-compliance in case of minimum wage laws (Basu, Chau, and Kanbur 2010) or workplace safety regulations (Johnson 2020), deliberate non-compliance could limit benefit provision. Second, mandates may not be strictly enforced, for example, due to pending lawsuits. Third, despite our best efforts, our classification of treatment status may include unavoidable imprecision due to the nature of the mandates and mapping legal specifics to data. As an example, in Connecticut, the mandate provides relief to firms that experience seasonal or transitional fluctuations in their workforce. This exemption may lead us to mis-classify some firms.

19 Recall that utilization is recorded for the past 12 months as of March in a given year. Thus, the first post-mandate year does not record the full 12 months, whereas the second does.

20 Recall that FFRCA included the first federal, but temporary, emergency sick leave provision for firms with fewer than 500 employees for reasons related to COVID-19. The provision went into effect April 1, 2020 and expired December 31, 2020 (it was later extended for initial non-users). The Act provided up to two weeks of sick leave and applied to all states. Further, select states expanded existing laws or passed new emergency sick leave legislation due to COVID-19; see A Better Balance (2021) and H.R.6201—Families First Coronavirus Response Act (2020); Jelliffe et al. (2021); and Andersen et al. (2023).

21 The industry and occupation classifications in the ACS are identical to the NCS. The NCS does not include information on education.

22 In further robustness checks, we control for the effective minimum wage; the results are very similar, and available on request.

23 Values not shown in any table or figure; more specific results are available upon request.

## Notes

*The editor in charge of this paper was Andrea Weber*.
