# Supplementary material for: Mandated Sick Pay: Coverage, Utilization, and Crowding-in
Source: J Eur Econ Assoc. 2025 Feb 22;23(5):1868–907. doi: 10.1093/jeea/jvaf008 (PMC12510734; doi:10.1093/jeea/jvaf008)
Supplement: jvaf008_Maclean_Pichler_Ziebarth_Online_Appendix [file jvaf008_maclean_pichler_ziebarth_online_appendix.pdf]

ONLINE APPENDIX TO "MANDATED SICK PAY"

Johanna Catherine Maclean
George Mason University

Stefan Pichler
University of Groningen

Nicolas R. Ziebarth
ZEW Mannheim
and University of Mannheim

Appendix A: Appendix

E-mail: jmaclea@gmu.edu (Maclean); s.pichler@rug.nl (Pichler);
nicolas.ziebarth@zew.de (Ziebarth)

Journal of the European Economic Association
Preprint prepared on 4 February 2025 using jeea.cls *v1.0.*

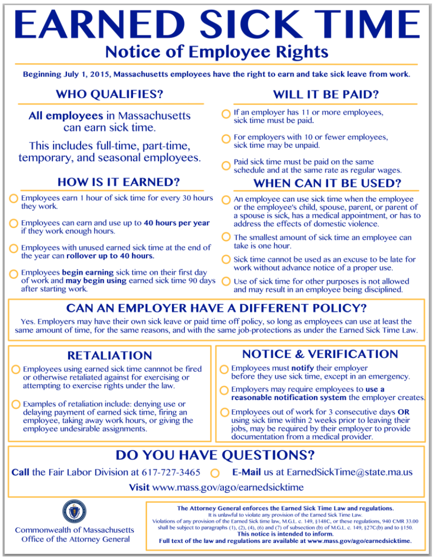

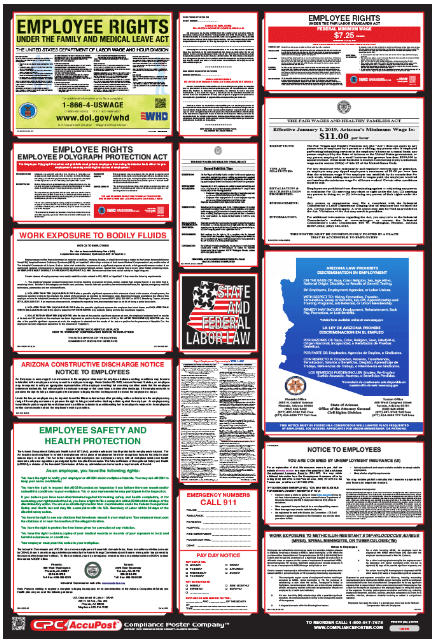

Figure A.1. Examples of legally required employee right notifications. Left figure shows an earned sick time poster from Massachusetts (Commonwealth of Massachusetts 2019). Right figure shows a general workplace poster that is compliant with notification requirements in Arizona (Industrial Commission of Arizona 2019). The Arizona poster includes all labor laws that firms are required to post at the workplace in Arizona.

Table A.1. Overview of state-level sick pay mandates in the U.S.

Region (1)	Law Passed (2)	Law Effective (3)	Content (4)
Washington D.C.	May 13, 2008	Nov 13, 2008	'qualified employees'; 1 hour of paid sick leave for every 43 hours, 90 days accrual period; up to 3 to 9 days depend. on Firm size; own sickness or family; no health care or restaurant employees
	Dec 18, 2013	Feb 22, 2014	extension to 20,000 temporary and tipped employees (retrosp. in Sep 2014)
Connecticut	July 1, 2011	Jan 1, 2012	full-time service sector employees at firms with >49 employees (20% of workforce); 1 hour for every 40 hours; up to 5 days; own sickness or family member, 680 hours accrual period (4 months)
California	September 19, 2014	July 1, 2015	all employees; 1 hour of paid sick leave for every 30 hours; minimum 24 hours; own sickness or family member; 90 days accrual period
Massachusetts	Nov 4, 2014	July 1, 2015	all employees at firms with >10 employees; 1 hour for every 40 hours; up to 40 hours; own sickness or family member; 90 days accrual period
Oregon	June 22, 2015	Jan 1, 2016	all employees at firms with >9 employees; 1 hour every 30 hours; 90 days accrual period; up to 40 hours; own sickness or family member
Vermont	March 9, 2016	Jan 1, 2017	employees w/ 18 hours/week & >20 weeks/year at firms with > 5 employees; 1 hour every 52 hours; up to 24 hours in 2017, 40 hours thereafter; own sickness or family member; underage employees and firms in first year exempt; some state employees & per diem employees in health care or long-term care facility exempt
Arizona	November 8, 2016	July 1, 2017	all employees; 1 hour for every 30 hours; up to 40 hours at firms with >14 employees, up to 24 hours <15 employees; own sickness or family member; firms can impose 90 day accrual period for new employees
Washington	Nov 8, 2016	Jan 1, 2018	all employees except those who are exempt from minimum wage law; 1 hour for every 40 hours; no cap but no more than 40 hours carry over; own sickness or family member; 90 day accrual for new employees
Maryland	Jan 12, 2018 (override veto by Governor)	Feb 11, 2018	employees w/ 12 hours/week at firms with > 14 employees (<15 employees 40 hours unpaid); 1 hour for every 30 hours; firms can cap at 64 hours accrual and 40 hours carry over; own sickness or family member, also for parental leave; certain groups exempt (e.g. temp. agency employees)
Rhode Island	Sept 28, 2017	July 1, 2018	all employees; 1 hour for every 35 hours; 24 hours in firms >17 (2018, 2019); 40 hours in firms >17 (2020+) own sickness or family member; 90-day accrual period;
New Jersey	May 2, 2018	Oct 28, 2018	all employees; 1 hour for every 30 hours up to 40 hours/year; per diem health care employees exempt own sickness or family member; 120 day accrual for new employees; preempts city laws

Overview of employer sick pay mandates in the U.S. (II).

**Region** (1)	**Law Passed** (2)	**Law Effective** (3)	**Content** (4)
Michigan	Dec 13, 2018 (weakened in lame duck session)	March 28, 2019	employees w/ 25 hours/week employed for 25 weeks at firms with > 49 employees; 1 hour for every 35 hours; government employees, certain railway and air carrier employees exempt; own sickness or family member; 90 day accrual for new employees
New York	April 3, 2020	Sep 30, 2020 (accrue) Jan 1, 2021 (take)	employees at firms with > 100 employees; up to 56 hours; <100 employees 40 hours (unpaid if <5 employees & < $1M in earnings); own sickness or family member; 1 hour per 30 hours of work; independent contractors and public employees exempt; accrual, use and bank can be limited to 48 hours
Colorado	July 14, 2020	immediately (covid-19) Jan 1 2021, Jan 1, 2022	all employees; 1 hour for every 30 hours; up to 48 hours p.a. supplemental sick leave when public health emergency; own sickness or family member; accrual, use and bank can be limited to 48 hours
New Mexico	April 1, 2021	July 1, 2022	all employees; some airline, railroad, government, tribe workers exempt; 1 hour for every 30 hours; own sickness or family member; use up to 64 hours p.a.
Minnesota	May 24, 2023	Jan 1, 2024	all employees with 80 hours/year; independent contractors exempt; building/construction exempt if covered by a CBA and clear waiver of requirements; 1 hour for every 30 hours; accrue and carry forward up to 80 hours; own sickness or family member

Source: A Better Balance (2024); National Partnership for Women & Families (2024), own collection, own illustration. Note: Nevada (Jan 1, 2020), Maine (Jan 1, 2023), and Illinois (Jan 1, 2024) passed paid time off mandates, requiring employers to allow employees to accrue general paid time off, without specific reason.

Appendix B: Appendix

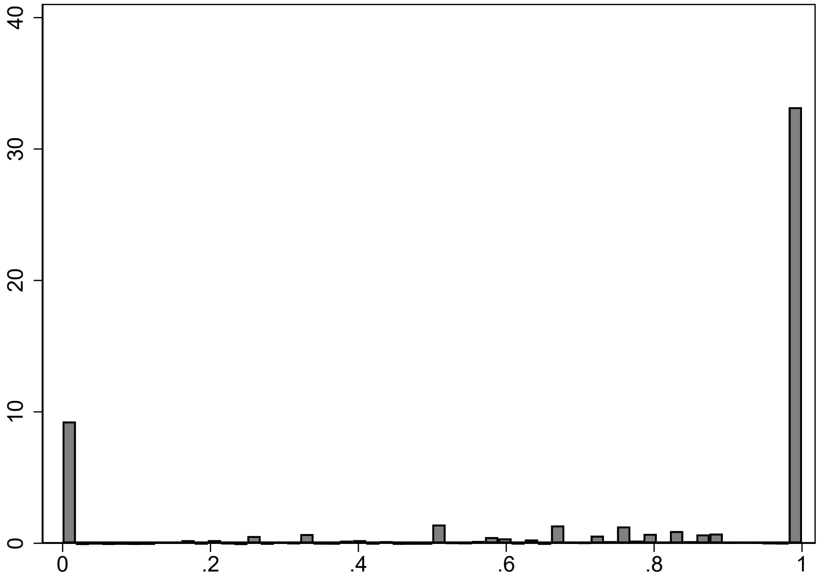

Figure B.1. Share of jobs within firms with voluntary sick pay. NCS data from 2009–2022 (Bureau of Labor Statistics 2023). Histogram conditions on states and years without sick pay mandates.

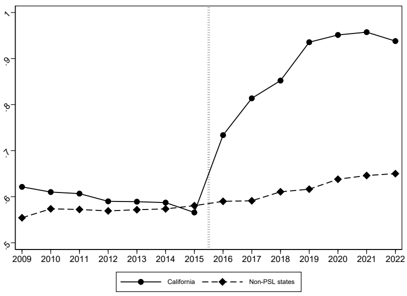

(a) California

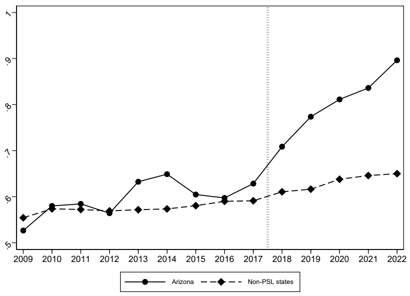

(b) Arizona

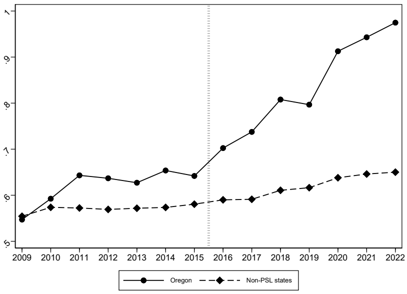

(c) Oregon

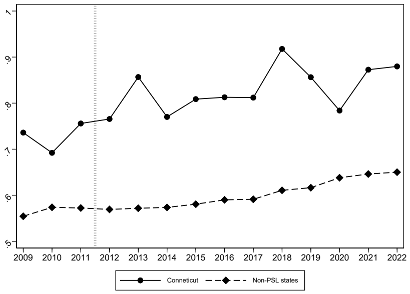

(d) Connecticut

Figure B.2. Treatment vs. control states: Unconditional time trends. NCS data from 2009–2022 (Bureau of Labor Statistics 2023). All graphs show unconditional trends of the share of jobs with paid sick leave by year and treatment state as indicated vs. the group of states without mandates. For more information about the sick pay reforms, see Table A.1.

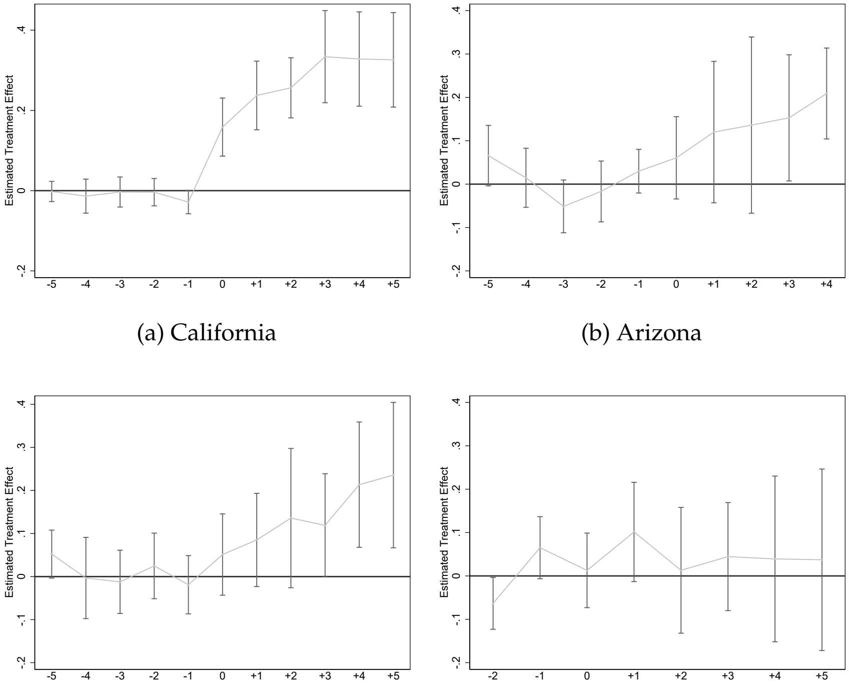

(a) California

(b) Arizona

(c) Oregon

(d) Connecticut

Figure B.3. Event studies by select treatment states. NCS data from 2009–2022 (Bureau of Labor Statistics 2023). All graphs show Callaway and Sant'Anna (2021) event studies. Standard errors are clustered at the state level and the gray bars depict 90% confidence intervals. Event studies include year and state fixed effects and only one treatment state as indicated (all other treated states are omitted). For more information about the sick pay reforms, see Table A.1.

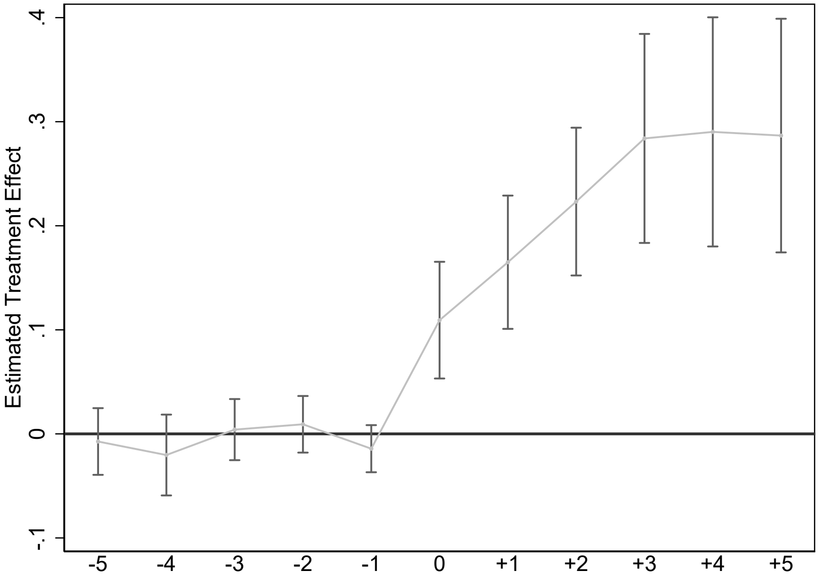

Figure B.4. Event studies including only states with full event time. NCS data from 2009–2022 (Bureau of Labor Statistics 2023). All graphs show Callaway and Sant'Anna (2021) event studies. Standard errors are clustered at the state level and the gray bars depict 90% confidence intervals. Event study includes year and state fixed effects only and conditions on states with full event times, that is, up to and including Oregon, see Table A.1.

Table B.1. Effect heterogeneity of mandates: industries and occupations.

**Outcome**	**Sick leave offered (1)**	**Paid sick hours taken (2)**	**Unpaid sick hours taken (3)**	**Sick leave costs per hour (5)**
Pretreatment mean: (in treated localities)	0.6262	17.9862	0.1354	0.3378
**Panel A: Industries**				
Construction				
Sick leave mandate	0.322***	7.931***	0.591***	0.136***
	(0.048)	(0.940)	(0.175)	(0.017)
Mean	0.4041	7.9482	0.1346	0.1348
Retail trade				
Sick leave mandate	0.234***	4.456***	0.355***	0.048***
	(0.020)	(0.502)	(0.044)	(0.010)
Mean	0.5069	9.8275	0.1914	0.1082
Admin, support, waste mngmt., and remed. services				
Sick leave mandate	0.348***	7.182***	0.167	0.098***
	(0.058)	(1.457)	(0.161)	(0.017)
Mean	0.3658	8.6642	0.0032	0.1334
Accommodation and food services				
Sick leave mandate	0.463***	4.093***	0.724**	0.044***
	(0.059)	(1.415)	(0.344)	(0.011)
Mean	0.1788	2.6916	0.0379	0.0241
**Panel B: Occupations**				
Food preparation and serving				
Sick leave mandate	0.451***	4.417***	0.832**	0.044***
	(0.054)	(0.992)	(0.330)	(0.009)
Mean	0.1784	3.2949	0.0610	0.0307
Sales and related				
Sick leave mandate	0.270***	3.833***	0.305***	0.048***
	(0.033)	(1.062)	(0.070)	(0.016)
Mean	0.5102	10.5490	0.1066	0.1373
Office and administrative				
Sick leave mandate	0.188***	5.102***	0.013	0.049***
	(0.027)	(1.277)	(0.123)	(0.018)
Mean	0.7453	22.6839	0.1499	0.2695
Transportation and material				
Sick leave mandate	0.222***	2.126	-0.102	-0.075
	(0.040)	(1.937)	(0.232)	(0.106)
Mean	0.5878	16.5359	0.3230	0.3158

Notes: NCS data from 2009-2022 (Bureau of Labor Statistics 2023).. Each cell stands for one Callaway and Sant'Anna (2021) model accounting for possible biases due to treatment dynamics and heterogeneity; ***, **, and * = statistically different from zero at the 1%, 5%, and 10% level. All models are weighted using NCS weights provided by the BLS. Standard errors clustered at the state level and reported in parentheses. All models have different firm-job observations, depending on industry and occupation. All models control for year and state fixed-effects (FE). For event studies, please see Figure 7.

Table B.2. Effect of sick pay mandates on hours worked vs. on paid leave.

	Annual hours			Total annual hours			Costs per hour		
	vacation (1)	holiday (2)	overtime (3)	worked (4)	paid leave (5)	paid (6)	wage (7)	health ins. (8)	non-production (9)
Pretreatment mean: *(in treated localities)*	68.6399	44.3836	47.9414	1665.4967	1804.7203	139.1737	24.8091	2.5877	0.6399
Panel A									
Sick leave mandate	5.048*	0.725	-5.396	1.076	11.073	10.042*	1.590*	0.206*	0.226**
	(2.698)	(1.838)	(6.344)	(22.286)	(26.297)	(5.484)	(0.850)	(0.107)	(0.096)
Year FE	X	X	X	X	X	X	X	X	
State FE	X	X	X	X	X	X	X	X	
Panel B									
Sick leave mandate	4.118***	0.062	-5.354*	-14.620**	-6.500	8.162***	1.391***	0.201***	0.202
	(1.364)	(0.748)	(3.237)	(6.403)	(6.370)	(2.272)	(0.496)	(0.064)	(0.169)
Year FE	X	X	X	X	X	X	X	X	
State FE	X	X	X	X	X	X	X	X	
Employee controls	X	X	X	X	X	X	X	X	

NCS data from 2009-2022 (Bureau of Labor Statistics 2023). Each cell stands for one Callaway and Sant’Anna (2021) model accounting for possible biases due to treatment dynamics and heterogeneity; ***, **, and * = statistically different from zero at the 1%, 5%, and 10% level. All models are weighted using NCS weights provided by the BLS. Employee controls: unionized job, part-time employment. Further, Panel B controls for paid time off mandates. Standard errors clustered at the state level and reported in parentheses. All models have 443,740 firm-job observations. For event studies, please see Figure 8.

Table B.3. Firm-level aggregation—Main treatment effects.

**Outcome**	**Sick leave offered (1)**	**Paid sick hours taken (2)**	**Unpaid sick hours taken (3)**	**Sick leave costs per hour (4)**
Pretreatment mean: *(in treated localities)*	0.7179	21.5191	0.1494	0.3987
Panel A				
Sick leave mandate	0.147***	2.907***	0.159***	0.042***
	(0.036)	(0.729)	(0.027)	(0.007)
Year FE	X	X	X	X
State FE	X	X	X	X
Panel B				
Sick leave mandate	0.150***	2.892***	0.172***	0.044***
	(0.035)	(0.673)	(0.025)	(0.008)
Year FE	X	X	X	X
State FE	X	X	X	X
Employee controls	X	X	X	X

Notes: NCS data from 2009-2022 (Bureau of Labor Statistics 2023). Each cell stands for one Callaway and Sant’Anna (2021) model accounting for possible biases due to treatment dynamics and heterogeneity; ***, **, and * = statistically different from zero at the 1%, 5%, and 10% level. All models are weighted using NCS weights provided by the BLS. Standard errors are clustered at the state level and reported in parentheses. Employee controls: unionized job, part-time employment. Further, Panel B controls for paid time off mandates. All models have 93,803 firm-year observations.

Table B.4. County-level aggregation—Main treatment effects.

Outcome	**Sick leave offered (1)**	**Paid sick hours taken (2)**	**Unpaid sick hours taken (3)**	**Sick leave costs per hour (4)**
Pretreatment mean: *(in treated localities)*	0.6140	17.4594	0.0680	0.2952
Panel A				
Sick leave mandate	0.185***	3.588***	0.224***	0.055**
	(0.048)	(1.331)	(0.066)	(0.023)
Year FE	X	X	X	X
State FE	X	X	X	X
Panel B				
Sick leave mandate	0.183***	3.438***	0.231***	0.054**
	(0.043)	(1.095)	(0.064)	(0.021)
Year FE	X	X	X	X
State FE	X	X	X	X
Job controls	X	X	X	X

Notes: NCS data from 2009-2022 (Bureau of Labor Statistics 2023). Yearly data at the county level. Each cell stands for one Callaway and Sant'Anna (2021) model accounting for possible biases due to treatment dynamics and heterogeneity; ***, **, and * = statistically different from zero at the 1%, 5%, and 10% level. Standard errors are clustered at the state level and reported in parentheses. Employee controls: unionized job, part-time employment. Further, Panel B controls for paid time off mandates. All models have 13,942 county-year observations.

Table B.5. State-level aggregation—Main treatment effects.

**Outcome**	**Sick leave offered (1)**	**Paid sick hours taken (2)**	**Unpaid sick hours taken (3)**	**Sick leave costs per hour (4)**
Pretreatment mean: *(in treated localities)*	0.6012	16.8011	0.1121	0.2983
Panel A				
Sick leave mandate	0.158***	3.169**	0.147***	0.061***
	(0.038)	(1.280)	(0.056)	(0.021)
Year FE	X	X	X	X
State FE	X	X	X	X
Panel B				
Sick leave mandate	0.205***	3.280***	0.068	0.060**
	(0.050)	(1.259)	(0.104)	(0.025)
Year FE	X	X	X	X
State FE	X	X	X	X
Job controls	X	X	X	X

Notes: NCS data from 2009-2022 (Bureau of Labor Statistics 2023). Yearly data at the county level. Each cell stands for one Callaway and Sant'Anna (2021) model accounting for possible biases due to treatment dynamics and heterogeneity; ***, **, and * = statistically different from zero at the 1%, 5%, and 10% level. Standard errors are clustered at the state level and reported in parentheses. Employee controls: unionized job, part-time employment. Further, Panel B controls for paid time off mandates. All models have 692 state-year observations.

Table B.6. Dropping employers below firm size mandate threshold.

Outcome	Sick leave offered (1)	Paid sick hours taken (2)	Unpaid sick hours taken (3)	Sick leave costs per hour (4)
Pretreatment mean: *(in treated localities)*	0.6302	18.2010	0.1384	0.3426
Panel A				
Sick leave mandate	0.203***	3.884***	0.171**	0.062***
	(0.046)	(1.033)	(0.073)	(0.023)
Year FE	X	X	X	X
State FE	X	X	X	X
Panel B				
Sick leave mandate	0.199***	3.621***	0.181**	0.058***
	(0.029)	(0.631)	(0.076)	(0.017)
Year FE	X	X	X	X
State FE	X	X	X	X
Employee controls	X	X	X	X

Notes: NCS data from 2009-2022 (Bureau of Labor Statistics 2023). Yearly data at the county level. Each cell stands for one Callaway and Sant'Anna (2021) model accounting for possible biases due to treatment dynamics and heterogeneity; ***, **, and * = statistically different from zero at the 1%, 5%, and 10% level. Standard errors are clustered at the state level and reported in parentheses. Employee controls: unionized job, part-time employment. Further, Panel B controls for paid time off mandates. All models have 437,099 firm-job observations. Here, firms below the mandate firm-size threshold (for states that differentiate mandates by firm size, see Table A.1) are dropped instead of assigned to the control group.

Table B.7. Keeping only California and untreated states.

**Outcome**	**Sick leave offered (1)**	**Paid sick hours taken (2)**	**Unpaid sick hours taken (3)**	**Sick leave costs per hour (4)**
Pretreatment mean: *(in treated localities)*	0.5954	16.9845	0.2474	0.3266
Panel A				
Sick leave mandate	0.278***	5.179***	0.134	0.076*
	(0.056)	(1.430)	(0.101)	(0.039)
Year FE	X	X	X	X
State FE	X	X	X	X
Panel B				
Sick leave mandate	0.268***	4.535***	0.149	0.068**
	(0.034)	(0.784)	(0.105)	(0.031)
Year FE	X	X	X	X
State FE	X	X	X	X
Employee controls	X	X	X	X
	(0.018)	(0.345)	(0.146)	(0.007)

Notes: NCS data from 2009-2022 (Bureau of Labor Statistics 2023). Each cell stands for one Callaway and Sant'Anna (2021) model accounting for possible biases due to treatment dynamics and heterogeneity; ***, **, and * = statistically different from zero at the 1%, 5%, and 10% level. All models are weighted using NCS weights provided by the BLS. Standard errors are clustered at the state level and reported in parentheses. Employee controls: unionized job, part-time employment. Further, Panel B controls for paid time off mandates. All models have 355,583 firm-job observations.

Table B.8. Using not yet treated as controls.

Outcome	**Sick leave offered (1)**	**Paid sick hours taken (2)**	**Unpaid sick hours taken (3)**	**Sick leave costs per hour (4)**
Pretreatment mean: *(in treated localities)*	0.6262	17.9862	0.1354	0.3378
Panel A				
Sick leave mandate	0.203***	3.958***	0.177***	0.066***
	(0.047)	(1.044)	(0.068)	(0.023)
Year FE	X	X	X	X
State FE	X	X	X	X
Panel B				
Sick leave mandate	0.197***	3.651***	0.187**	0.062***
	(0.029)	(0.620)	(0.073)	(0.017)
Year FE	X	X	X	X
State FE	X	X	X	X
Employee controls	X	X	X	X

Notes: NCS data from 2009-2022 (Bureau of Labor Statistics 2023). Yearly data at the county level. Each cell stands for one Callaway and Sant’Anna (2021) model accounting for possible biases due to treatment dynamics and heterogeneity; ***, **, and * = statistically different from zero at the 1%, 5%, and 10% level. Standard errors are clustered at the state level and reported in parentheses. Employee controls: unionized job, part-time employment. Further, Panel B controls for paid time off mandates. All models have 443,740 firm-job observations.

Table B.9. Drop Covid-19 years (2020–2022)

**Outcome**	**Sick leave offered (1)**	**Paid sick hours taken (2)**	**Unpaid sick hours taken (3)**	**Sick leave costs per hour (4)**
Pretreatment mean: *(in treated localities)*	0.6244	17.9040	0.1389	0.3371
Panel A				
Sick leave mandate	0.181***	2.415***	0.324***	0.046**
	(0.042)	(0.781)	(0.120)	(0.019)
Year FE	X	X	X	X
State FE	X	X	X	X
Panel B				
Sick leave mandate	0.177***	2.168***	0.339***	0.044***
	(0.041)	(0.603)	(0.060)	(0.009)
Year FE	X	X	X	X
State FE	X	X	X	X
Employee controls	X	X	X	X

Notes: NCS data from 2009-2022 (Bureau of Labor Statistics 2023). Yearly data at the county level. Each cell stands for one Callaway and Sant'Anna (2021) model accounting for possible biases due to treatment dynamics and heterogeneity; ***, **, and * = statistically different from zero at the 1%, 5%, and 10% level. Standard errors are clustered at the state level and reported in parentheses. Employee controls: unionized job, part-time employment. Further, Panel B controls for paid time off mandates. All models have 381,911 firm-job observations.

References

Bureau of Labor Statistics (2023). *National Compensation Survey*. https://www.bls.gov/ebs/publications/september-2022-landing-page-employee-benefits-in-the-united-states-march-2022.htm, retrieved July 19, 2023.

Callaway, Brantly and Pedro HC Sant'Anna (2021). "Difference-in-differences with multiple time periods." *Journal of Econometrics*, 225(2), 200–230.
