# Supplementary material for: Mandated Sick Pay: Coverage, Utilization, and Crowding-in
Source: J Eur Econ Assoc. 2025 Feb 22;23(5):1868–907. doi: 10.1093/jeea/jvaf008 (PMC12510734; doi:10.1093/jeea/jvaf008)
Supplement: jvaf008_Maclean_Pichler_Ziebarth_Replication_Files [file jvaf008_maclean_pichler_ziebarth_replication_files.pdf]

MANDATED SICK PAY: COVERAGE, UTILIZATION, AND CROWDING-IN

Johanna Catherine Maclean
George Mason University and NBER

Stefan Pichler
University of Groningen

Nicolas R. Ziebarth
ZEW Mannheim and University of Mannheim

The replication package for this paper is available at https://zenodo.org/records/14500757. The authors were granted an exemption to publish their data because either access to the data is restricted or the authors do not have the right to republish them. However, the authors provided a synthetic dataset that allows to run their codes, although these data are not designed to reproduce the results in the paper. The JEEA checked the codes using the synthetic data for their ability to generate all tables and figures in the paper and any approved online appendices.

E-mail: jmaclea@gmu.edu (Maclean); s.pichler@rug.nl (Pichler); nicolas.ziebarth@zew.de (Ziebarth)

Journal of the European Economic Association
Preprint prepared on 9 January 2025 using jeea.cls *v1.0.*
